# mLearning in the Democratic Republic of the Congo: A Mixed-Methods Feasibility and Pilot Cluster Randomized Trial Using the Safe Delivery App

**DOI:** 10.9745/GHSP-D-18-00275

**Published:** 2018-12-27

**Authors:** Nancy E. Bolan, Larry Sthreshley, Bernard Ngoy, Faustin Ledy, Mano Ntayingi, Davis Makasy, Marie-Claude Mbuyi, Gisele Lowa, Lynne Nemeth, Susan Newman

**Affiliations:** a College of Nursing, Medical University of South Carolina, Charleston, SC, USA.; b IMA World Health, Kinshasa, Democratic Republic of the Congo.; c Pathfinder International, Kinshasa, Democratic Republic of the Congo.

## Abstract

**Background:**

Substandard delivery care has been widely documented as a major cause of maternal mortality in health facilities globally. Health worker learning via mobile devices is increasing rapidly; however, there is little evidence of mLearning effectiveness. This study sought to determine the feasibility, acceptability, and potential effect of the Safe Delivery App (SDA) on health workers' practices in basic emergency obstetric and newborn care (BEmONC) in the Democratic Republic of the Congo (DRC). The Theoretical Domains Framework was used to guide this research.

**Methods:**

Eight BEmONC facilities in central DRC were randomized to either an mLearning intervention or to standard practice (control). Maternal and newborn health workers in intervention facilities (n=64) were trained on the use of smartphones and the French version of the SDA. The SDA is an evidence-based BEmONC training resource with visual guidance using animated videos and clinical management instructions developed by the Maternity Foundation and the Universities of Copenhagen and Southern Denmark. Knowledge on postpartum hemorrhage (PPH) and neonatal resuscitation (NR) and self-confidence in performing 12 BEmONC procedures were assessed at baseline and at 3 months post-intervention. Eighteen qualitative interviews were conducted with app users and key stakeholders to assess feasibility and acceptability of mLearning and the use of the SDA. Maternal mortality was compared in intervention and control facilities using a smartphone-based Open Data Kit (ODK) data application. One smartphone with SDA and ODK was entrusted to intervention facilities for the study period, whereas control facilities received smartphones with ODK only.

**Results:**

The analysis included 62 heath workers. Knowledge scores on postpartum hemorrhage and neonatal resuscitation increased significantly from baseline among intervention participants compared with controls at 3 months post-intervention (mean difference for PPH knowledge, 17.4 out of 100; 95% confidence interval [CI]=10.7 to 24.0 and 19.4 for NR knowledge; 95% CI=11.4 to 27.4), as did self-confidence scores on 12 essential BEmONC procedures (mean difference, 4.2 out of 48; CI=0.7 to 7.7). Increases were unaffected by health worker cadre and previous smartphone use. Qualitative interviews supported the feasibility and acceptability of the SDA and mLearning, and the potential for it to impact maternal and neonatal mortality in the DRC.

**Conclusion:**

Use of the Safe Delivery App supported increased health worker knowledge and self-confidence in the management of obstetric and newborn emergencies after 3 months. SDA and mLearning were found to be feasible and acceptable to health workers and key stakeholders in the DRC.

## Abstract (precis)

Health worker knowledge and self-confidence in basic emergency obstetric and newborn care (BEmONC) increased significantly 3 months after introduction of the Safe Delivery App in intervention facilities compared with controls.

Résumé en français à la fin de l'article. Le texte complet de l'article est aussi disponible en français.

## INTRODUCTION

Health worker clinical performance is often inadequate in low- and middle-income countries (LMICs).^1^ Substandard services in delivery and emergency obstetric and newborn care (EmONC) have been widely documented as a major cause of maternal and newborn mortality in health facilities globally.^2^ Worldwide, approximately 830 women die daily from preventable causes related to pregnancy and childbirth,^3^ and almost 2 million newborns die in the first week of life every year.^4^ LMICs accounted for approximately 99% (302,000) of global maternal deaths in 2015, with sub-Saharan Africa alone accounting for about 66% of deaths (201,000).^5^

The Democratic Republic of the Congo (DRC), the largest country in sub-Saharan Africa, has one of the highest maternal mortality ratios in Africa (846 maternal deaths per 100,000 live births).^6^ A woman's lifetime risk of maternal death, or the probability that a 15-year-old woman will eventually die from a maternal cause, is estimated to be 1 in 24 in the DRC, compared with 1 in 3,300 in high-income countries.^5^ Neonatal deaths, or deaths before 28 days of life, are estimated at 29 per 1,000 live births in the DRC.^4^ Worldwide, disparities in maternal and child health outcomes largely reflect inequalities in access to quality health services.^3^

Deficits in health worker knowledge and skills are linked to suboptimal patient outcomes in low-resource settings.^7^^–^^9^ Maternal care providers demonstrate low levels of EmONC knowledge, despite varying years of provider experience, and poor clinical management skills of postpartum hemorrhage (PPH).^2^^,^^10^^–^^12^ PPH is the leading cause of maternal mortality worldwide,^13^ and PPH management is 1 of the 7 “signal functions” of basic EmONC (BEmONC), or key medical interventions that must be provided by all skilled birth attendants. An outreach gap exists wherein health workers in peripheral health facilities are not properly trained to manage obstetric emergencies.^14^ Additionally, in low-volume settings, emergencies do not occur sufficiently often for providers to become experienced in obstetric complication management.^15^

A basic strategy for changing health worker behavior and strengthening clinical performance is promoting continuing education (CE) or continuous professional development.^16^ However, for many health workers, access to relevant up-to-date learning opportunities is difficult or impossible, particularly in hard-to-reach or peripheral settings where maternal and newborn mortality are highest.^10^^,^^17^^,^^18^ However, the availability and use of mobile phones is increasing rapidly in LMICs,^19^ as is learning via mobile devices or mLearning. Given the costs and logistical challenges of providing in-person, conventional CE training programs peripherally, the use of mobile phones and other mobile electronic devices holds promise as new mechanisms to reach more remote health care workers with up-to-date information.^20^ Most of the studies examining mLearning, however, are of poor methodological quality and few have evaluated the effects on client health outcomes.^21^^–^^23^

The use of mobile phones holds promise to reach more remote health care workers with up-to-date information.

This mixed-methods feasibility and pilot cluster randomized controlled trial (RCT) sought to determine the feasibility, acceptability, and potential impact of a recently developed evidence-based mLearning training tool, known as the Safe Delivery App (SDA), on knowledge, self-confidence, and practice of facility-based health workers in maternal and newborn health in the DRC. The trial also sought to refine intervention delivery in the DRC and strengthen study procedures required to conduct a robust large-scale trial in the future. The Theoretical Domains Framework guided this study, which views health professional behavior change as key to increasing the uptake of evidence into health care practice.^24^

## METHODS

### Study Design

This feasibility pilot study was a cluster RCT with the health care facility as the unit of randomization. The study followed the Consolidated Standards of Reporting Trials (CONSORT) guidelines for reporting pilot and feasibility trials.^25^ Using mixed-methods convergent parallel design,^26^ the principal investigator (PI) conducted qualitative semistructured interviews with app users and key stakeholders.^27^ Additionally, selected patient outcomes were compared pre- and post-intervention. The DRC Institutional Review Board (IRB) housed at the Protestant University of Congo (UPC) provided ethical clearance for the study in April 2017, as did the Medical University of South Carolina (USA) IRB.

### Setting

The study took place over 3 months (April–July 2017) in 2 health zones (Alunguli and Kindu) in the province of Maniema, an under-resourced area in central-eastern DRC with weak infrastructure and some of the poorest maternal and newborn health outcomes in the country.^6^ Ten health care facilities constituted sites eligible for cluster randomization owing to their being accessible by vehicle and being designated as EmONC centers supported by the Access to Primary Health Care Project (ASSP). ASSP, led by IMA World Health (IMA), an international NGO, is a health systems strengthening and primary care redevelopment project funded by the UK government. The project is carried out in collaboration with the Congolese government and an array of local and international partners to revitalize the country's health system in targeted health zones, fight disease, and improve key health indicators, particularly related to maternal and child mortality.^28^ As designated EmONC centers, the 10 facilities (1 hospital and 4 health centers per zone) have received EmONC commodities and equipment, and personnel have participated in EmONC trainings.

### Randomization

Identified facilities were stratified by type into hospital or health center categories (Figure). In the hospital category, 1 facility was selected randomly for intervention, using an urn filled with labeled papers, from the matched group of 2 facilities. One health center was excluded due to being non-functional. In the health center category, 3 centers were chosen randomly from among the 7 matched health centers for intervention and 3 for control, giving a total of 4 intervention and 4 control facilities (N=8).

**FIGURE:**
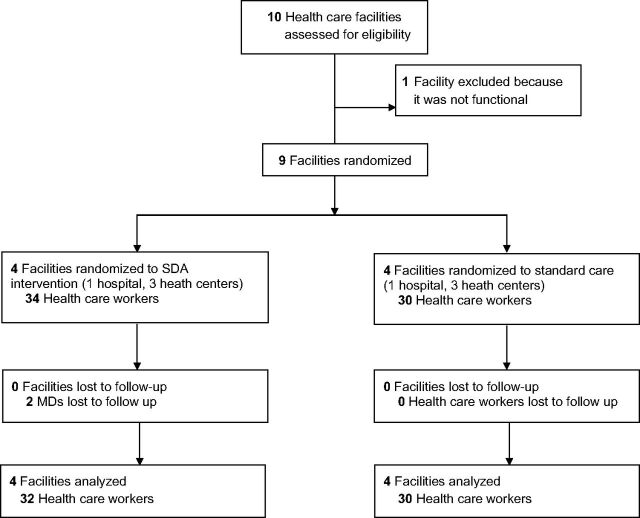
CONSORT Flow Diagram Abbreviations: CONSORT, Consolidated Standards of Reporting Trials; MD, medical doctor.

### Participants

Medical doctors (MDs), nurses, and midwives working in the selected facilities who manage deliveries and newborn care were invited to participate in the study. The study population included 64 health care workers at the 8 selected health care facilities (Figure). Attrition included 2 individuals (MDs) in the intervention group who completed the pretest but were unable to participate in the posttest due to ill health. For this mixed-methods study, the PI conducted qualitative semistructured interviews with 2 categories of professionals for a cumulative total of 18 interviews. The first category of professionals consisted of 10 key stakeholders in Kinshasa (national capitol) and Kindu (capitol of Maniema Province), and the second category comprised 8 app users. For key stakeholders, the researcher used “snowball sampling,” which relies on the personal networks of the persons the researcher taps into for referrals to other key persons.^29^ Key stakeholders included educational, policy, program and health service leaders in the field. App users were identified as a convenience sample from among users trained on the app from the study facilities in Kindu.^26^ All study participants provided verbal consent after they were informed about the purposes of the study during a site visit by the research team and were given IRB-approved written information (“Information for Participants Sheet”), assuring the confidentiality of all information obtained during the study and informing them of their right to withdraw from the study at any time without any effect on their employment status. The study participants and the clinic staff were not masked because the intervention required overt participation. Facilities were randomized rather than individuals to avoid contamination among health care workers in the same facility.^14^

The study included medical doctors, nurses, and midwives working in the DRC who manage deliveries and newborn care.

### Intervention

The SDA is a training tool and job aid developed by the Maternity Foundation, University of Copenhagen, and the University of Southern Denmark. It was designed to reinforce the capability and confidence of health care workers in low-income countries on how to manage basic obstetric and neonatal emergencies. The content of the app is based on global clinical BEmONC guidelines and has been validated by an international group of global health experts.^15^ The SDA can be downloaded free of charge for iPhone at https://itunes.apple.com/dk/app/safe-delivery/id985603707?mt=8 and for Android at https://play.google.com/store/apps/details?id=dk.maternity.safedelivery.

The SDA conveys knowledge and skills via animated videos and instructions on key procedures. It also contains information on essential drugs for BEmONC. All features and functions are designed for low-literacy, low-income settings and work completely offline once downloaded. The 10 instruction films include the 7 signal functions of BEmONC as well as 3 additional essential procedures (infection prevention, management of infection in newborns, and active management of the third stage of labor). In this study, the French version of the SDA was pre-downloaded to Android smartphones in the DRC capital, Kinshasa, due to poor Internet connectivity in the pilot region (Kindu, Maniema), and 1 smartphone was allocated per facility. An Open Data Kit (ODK) data collection instrument, purposefully designed by the PI and study authors for this study, was also loaded onto the smartphones to collect information on BEmONC vital statistics and signal function execution, beyond what was normally captured in the District Health Information System 2 (DHIS 2) (to be referred to as the health information system, or HIS), and was to be entered manually by facility staff daily.

The Safe Delivery App conveys knowledge and skills via animated videos and instructions on key BEmONC procedures.

Staff in participating facilities received explanation of the nature and purpose of the trial. Intervention health care workers received a half-day training session on the use of the smartphone, SDA, and ODK, with joint app video viewing and discussion. At the non-intervention health care facilities, the health care workers provided standard care without the assistance of the SDA. However, training was conducted for the smartphone-based ODK data collection, as data were collected at control facilities in the same manner as at intervention facilities during the study period. To ensure equal possibilities to provide standard care, the availability of a minimum package of drugs and equipment was ensured by ASSP in both groups of facilities. For the 3-month study period, the smartphone with SDA was available to all maternity providers at the intervention facilities. Solar panel battery chargers were given to all 8 facilities with the smartphones to ensure consistent ability to charge. Providers were instructed to use the SDA as often as they wished and that the phone should be made available to the team on duty at all times. Ministry of Health supervisors were tasked with visiting intervention and control facilities weekly to remind providers to use the app and/or the ODK.

### Theoretical Framework

The Theoretical Domains Framework, which positions health professional behavior change as key to increasing the uptake of evidence into health care practice, was used to guide this research.^24^ The initial aim of this framework was to simplify and integrate a number of behavior change theories to provide a theoretical lens through which to view the cognitive, affective, social, and environmental influences on provider behavior.^30^^–^^31^ Explanatory constructs from 33 theories of behavior change were reduced and grouped into 14 theoretical construct domains, each of which consists of a grouping of theoretical constructs, which are proposed as potential mediators of behavior change (Table 1).^30^^–^^31^ The Theoretical Domains Framework provides a useful conceptual basis for assessing implementation problems of evidence-based care and understanding provider behavior-change processes.^24^ In this research, the Theoretical Domains Framework influenced the design of interview questions to explore the specific content of these domains in relation to barriers and facilitators to the use of the SDA, mLearning, and CE implementation in the DRC. It was also used as the coding framework for analysis.

**TABLE 1.** The Theoretical Domains Framework With Definitions and Component Constructs^30^

Domain	Construct	Domain	Construct
**1. Knowledge** (an awareness of the existence of something)	Knowledge (including knowledge of condition/scientific rationale) Procedural knowledge Knowledge of task environment	**8. Intentions** (a conscious decision to perform a behavior or a resolve to act in a certain way)	Stability of intentions Stages of change model Transtheoretical model and stages of change
**2. Skills** (an ability or proficiency acquired through practice)	Skills Skill development Competence Ability Interpersonal skills Practice Skill assessment	**9. Goals** (mental representations of outcomes or end states that an individual wants to achieve)	Goals (distal/proximal) Goal priority Goal/target setting Goals (autonomous/controlled) Action planning Implementation intention
**3. Social/professional role and identity** (a coherent set of behaviors and displayed personal qualities of an individual in a social or work setting)	Professional identity Professional role Social identity Identity Professional boundaries Professional confidence Group identity Leadership Organizational commitment	**10. Memory, attention, and decision processes** (the ability to retain information, focus selectively on aspects of the environment, and choose between 2 or more alternatives)	Memory Attention Attention control Decision making Cognitive overload/tiredness
**4. Beliefs about capabilities** (acceptance of the truth, reality, or validity about an ability, or talent that a person can put to constructive use)	Self-confidence Perceived competence Self-efficacy Perceived behavioral control Beliefs Self-esteem Empowerment Professional confidence	**11. Environmental context and resources** (any circumstance of a person's situation or environment that discourages or encourages the development of skills and abilities, independence, social competence, and adaptive behavior)	Environmental stressors Resources/material resources Organizational culture/climate Salient events/critical incidents Person–environment interaction Barriers and facilitators
**5. Optimism** (the confidence that things will happen for the best or that desired goals will be attained)	Optimism Pessimism Unrealistic optimism Identity	**12. Social influences** (those interpersonal processes that can cause individuals to change their thoughts, feelings, or behaviors)	Social pressure Social norms Group conformity Social comparisons Group norms Social support Power Intergroup conflict Alienation Group identity Modeling
**6. Beliefs about consequences** (acceptance of the truth, reality, or validity about outcomes of a behavior in a given situation)	Beliefs Outcome expectancies Characteristics of outcome expectancies Anticipated regret Consequents	**13. Emotion** (a complex reaction pattern, involving experiential, behavioral, and physiological elements, by which the individual attempts to deal with a personally significant matter/event)	Fear Anxiety Affect Stress Depression Positive/negative affect Burn-out
**7. Reinforcement** (increasing the probability of a response by arranging a dependent relationship, or contingency, between the response and a given stimulus)	Rewards (proximal/distal, valued/not valued, probable/improbable) Incentives Punishment Consequents Reinforcement Contingencies Sanctions	**14. Behavioral regulation** (anything aimed at managing or changing objectively observed or measured actions)	Self-monitoring Breaking habit Action planning

The Theoretical Domains Framework reduces and groups explanatory constructs from 33 theories of behavior change into 14 theoretical construct domains.

### Outcomes and Measures

The primary outcomes of the pilot SDA trial were self-confidence and knowledge scores by the health care workers. Self-confidence and knowledge data collection instruments were developed, tested (in English), and translated into French by the Maternity Foundation in Copenhagen. Reliability and validity measures have not yet been published for these measures; this study will contribute to the assessment of the measures. Self-confidence scores were assessed for 12 essential BEmONC services. Knowledge scores were assessed for 2 key BEmONC services, management of PPH and neonatal resuscitation (NR) at baseline and at 3 months post-intervention. Additionally, baseline demographic characteristics were collected for the health workers in intervention and control groups.

Births, maternal deaths, obstetric complications, and execution of BEmONC signal functions were assessed in intervention and control clusters post-intervention using a smartphone-based ODK application designed for this study by the researchers and piloted with the SDA, as part of an examination of study procedures for a future adequately powered RCT. ODK-generated data were compared with hand-collected statistical data from health facility registers and with HIS data.

Feasibility and acceptability of the SDA were assessed through qualitative semistructured interviews with app users. Key stakeholder perspectives on the use of mLearning more broadly in the DRC were also assessed.

### Data Collection

Data collection was conducted in parallel in the intervention and control facilities using the same methods at baseline and 3 months after the training intervention. Measures were taken prior to the training of facility-based providers on the SDA and included:

- Demographic data of participant health workers (pre-intervention only)
- Provider self-rated self-confidence in handling 12 essential procedures of BEmONC
- Provider knowledge of 2 key BEmONC services: management of PPH and NR

All data were collected on paper in a classroom setting; knowledge scoring was provided by the SDA. Results were entered in Microsoft Excel and subsequently transferred and analyzed in SPSS (version 23). Three months after the SDA introduction, self-confidence and knowledge were measured a second time in the classroom using the same data collection instruments.

The PI developed 2 qualitative interview guides for the 2 qualitative target groups (SDA users and key stakeholders) using the theoretical construct domains of the Theoretical Domains Framework^31^ to guide the questions (Table 1). Semistructured interviews were audio recorded by the PI with 8 SDA users and 10 key stakeholders after the 3-month study period. SDA users were asked about the feasibility and acceptability of using the SDA and barriers and facilitators to its use. Key stakeholders were asked about the feasibility and acceptability of the use of mLearning and CE in the DRC more broadly, as well as barriers and facilitators to the implementation of CE.

Facility-based reporting of selected health outcomes collected with the use of the ODK was compared with data reported in the HIS and with data collected by hand-review of health facility registers by the PI. Data were collected by hand at baseline for the 3 months prior to the intervention and then at 3 months post-intervention for comparison. The ODK app was developed for this research study and piloted during the study period in the 8 intervention and control facilities. The ODK data were entered by the health workers into mobile phones provided by the project immediately post-delivery/event and were available online (after uploading) for consultation from any location.

### Statistical Analysis

Descriptive summary statistics were analyzed on demographic data including age, gender, profession, educational level, years of experience, number of deliveries performed in the past month, and previous use of smartphone. Given that this was a feasibility study, power calculations were not made in choosing the sample size for the pilot trial. However, the study team did gear the sampling strategy to achieve a minimum sample size of 30 for both intervention and control groups to support the use of parametric statistical tests.

T-tests examined within-subject differences on test scores pre- and post-intervention, (where the dependent variable was the score on self-confidence and knowledge tests within the intervention and control groups) and between-group differences in change in self-confidence and knowledge (where the dependent variable was the mean difference in change on scores for the 2 groups). Confidence intervals (CIs) and effect size were calculated. To test for potential confounding, between-group differences were calculated to examine the role of gender on test scores and the role of previous smartphone use. One-way analysis of variance (ANOVA) was used to examine test scores analyzed by the 3 health professional cadres (nurses, midwives, and MDs) across both intervention and control groups. The criterion for significance for all analyses was set at *P*<.05. All data were entered into Microsoft Excel and analysis was performed using SPSS (version 23).

### Qualitative Analyses

Data coding for both target groups was carried out deductively by the PI, using the 14 domains from the Theoretical Domains Framework (Table 1) as the coding framework for content analysis, in order to interpret meaning from the content of the qualitative data.^30^^–^^31^

Quantitative and qualitative data were interpreted and merged together, noting both the quantitative statistical results and qualitative quotes or themes that supported or refuted the quantitative results.^27^

## RESULTS

### Quantitative Data

#### Background Characteristics

The analysis included 62 health care workers: 32 in intervention and 30 in control groups. Table 2 shows that the participating health care workers included 26 clinical nurses and midwives (81.3%) in intervention groups and 20 (66.6%) in control groups; the remaining workers were medical doctors (18.8% intervention and 33.3% control, respectively). The average age was similar, 41.3 years and 44.2 years in the intervention and control groups, respectively. There were more women in the intervention groups (n=26, 81.3%) than the control groups (n=11, 36.7%). The control groups had less delivery experience than the intervention groups, with 13 workers (43.3%) conducting 5 or fewer deliveries during the previous month and 10 (33.3%) conducting more than 10 deliveries, in comparison with 5 (15.6%) and 21 (65.6%) workers, respectively, in the intervention groups. Similarly, 12 workers (40.0%) had more than 10 years of experience in the profession in the control groups, compared with 17 (53.1%) in the intervention groups. Fourteen intervention health care workers (43.8%) and 9 control health workers (30.0%) had tried using a smartphone before the study.

**TABLE 2.** Demographic Characteristics of Study Groups^a^

	Intervention	Control
	(n=32)	(n=30)
**Age, years, mean**	41.3	44.2
**Gender, No. (%)**		
Male	6 (18.8)	19 (63.3)
Female	26 (81.3)	11 (36.7)
**Professional cadre, No. (%)**		
Nurses	22 (68.8)	14 (46.6)
Midwives	4 (12.5)	6 (20.0)
MDs	6 (18.8)	10 (33.3)
**Years of experience in profession, No. (%)**		
1–5	3 (9.4)	6 (20.0)
6–10	12 (37.5)	12 (40.0)
>10	17 (53.1)	12 (40.0)
**No. of deliveries past month, No. (%)**		
0–5	5 (15.6)	13 (43.3)
6–10	6 (18.8)	7 (23.3)
>10	21 (65.6)	10 (33.3)
**Experience with smartphone, No. (%)**		
Tried using one	14 (43.8)	9 (30.0)
Never tried using one	18 (56.3)	21 (70.0)

Abbreviation: MD, medical doctor.

a The intervention and control group each comprised 4 health care facilities.

#### Baseline Knowledge and Self-Confidence Scores

Mean knowledge scores for PPH management were similar at baseline for health workers in the intervention and control groups at 47.8 (standard deviation [SD]=16.8) and 47.5 (SD=14.7), respectively, out of 100 total points (Table 3). BEmONC self-confidence mean scores were also similar at baseline in intervention and control groups at 30.3 (SD=8.7) and 31.4 (SD=10.8), respectively, out of 48 total points. In contrast, mean baseline NR knowledge scores were lower among health care workers in the intervention group (40.8, SD=17.5) compared with the control group (50.9, SD=16.6) (out of 100 points).

**TABLE 3.** Within- and Between-Subject Differences in Mean Knowledge and Self-Confidence Scores Pre-and-Post Intervention

	Intervention	Control	Difference Between Intervention and Control	95% CI	*P* Value	Cohen's d
	(n=32)	(n=30)				
**PPH knowledge scores (out of 100)**						
Pre, mean (SD)	47.8 (16.8)	47.5 (14.7)	0.3	(−7.8, 8.3)	.95	
Post, mean (SD)	66.7 (14.8)	49.1 (15.6)				
Pre-post difference, mean (SD)	18.9 (14.6)	1.6 (11.4)	17.4	(10.7, 24.0)	<.001	1.6
95% CI for pre-post difference	(13.7, 24.2)	(−2.6, 5.8)				
*P* value for pre-post difference	<.001	.46				
Cohen's d for pre-post difference	1.2					
**NR knowledge scores (out of 100)**						
Pre, mean (SD)	40.8 (17.5)	50.9 (16.6)	−10.1	(−18.7, −1.4)	.02	-0.6
Post, mean (SD)	57.7 (15.3)	48.3 (17.3)				
Pre-post difference, mean (SD)	16.8 (14.6)	−2.5 (16.9)	19.4	(11.4, 27.4)	<.001	1.2
95% CI for pre-post difference	(11.6, 22.1)	(−3.8, 3.8)				
*P* value for pre-post difference	<.001	.42				
Cohen's d for pre-post difference	1.0					
**BEmONC self-confidence scores (out of 48)**						
Pre, mean (SD)	30.3 (8.7)	31.4 (10.8)	−1.1	(−6.1, 3.9)	.66	
Post, mean (SD)	34.0 (8.9)	30.9 (8.7)				
Pre-post difference, mean (SD)	3.8 (6.6)	−0.4 (7.2)	4.2	(0.7, 7.7)	.02	0.6
95% CI for pre-post difference	(1.4, 6.2)	(−8.6, 2.3)				
*P* value for pre-post difference	.003	.74				
Cohen's d for pre-post difference	0.4					

Abbreviations: BEmONC, basic emergency obstetric and newborn care; CI, confidence interval; NR, neonatal resuscitation; PPH, postpartum hemorrhage, SD, standard deviation.

#### Pre-Post Differences in Knowledge and Self-Confidence Scores

We found a significant association between the SDA intervention and health care workers' knowledge on both PPH and NR knowledge, as well as on BEmONC self-confidence, 3 months after baseline (Table 3). The mean increase in PPH knowledge from pre- to post-test was statistically significantly larger in the intervention group compared with the control group (18.9, SD=14.6 vs 1.6, SD=11.4, respectively; *P*<.001). Similarly, the mean increase in NR knowledge from pre- to post-test in the intervention group was statistically significantly larger compared with the increase in the control group (16.8, SD=14.6 vs −2.5, SD=16.9, respectively; *P*<.001), despite lower baseline scores in the intervention group. Overall self-confidence scores on 12 essential EmONC procedures also significantly improved compared with those of controls after 3 months (mean difference, 4.2 out of 48; CI=0.7 to 7.7; *P*=.02). Significant differences in the self-confidence of intervention participants were found pre- and post-test on 5 essential BEmONC procedures out of 12: manual vacuum aspiration, preeclampsia/eclampsia, prolonged labor, PPH, and manual placenta removal (Table 4).

**TABLE 4.** Differences in BEmONC Self-Confidence Scores (on a Scale of 4 Points) Among the Intervention Group, Pre- and Post-Intervention

BEmONC Procedures	Pre-	Post-	Pre-Post Difference	*P* Value
	Mean (SD)	Mean (SD)		
MVA	2.1 (1.3)	3.0 (1.2)	0.88	.003
D&C	2.7 (1.2)	2.6 (1.3)	−0.03	.90
Preeclampsia/eclampsia	2.0 (1.2)	2.4 (1.4)	0.41	.03
AMTSL	3.5 (0.7)	3.7 (0.5)	0.22	.09
Prolonged labor	2.7 (1.1)	3.1 (0.8)	0.47	.007
Vacuum extraction	1.7 (1.4)	1.8 (1.3)	0.19	.45
PPH	2.6 (1.2)	3.1 (0.9)	0.50	.03
Manual placental removal	2.8 (1.0)	3.1 (1.1)	0.31	.04
Septicemia	2.2 (1.2)	2.5 (1.3)	0.34	.08
NR	3.0 (0.8)	3.0 (0.9)	0.00	1.00
Danger signs in newborns	2.6 (1.0)	2.9 (1.0)	0.31	.11
Severe infection of newborn	2.4 (1.1)	2.6 (1.0)	0.19	.31

Abbreviations: AMTSL, active management of the third stage of labor; D&C, dilation and curettage; MVA, manual vacuum aspiration; NR, neonatal resuscitation; PPH, postpartum hemorrhage.

The Safe Delivery App had a significant effect on health workers' knowledge of postpartum hemorrhage and neonatal resuscitation, and on their BEmONC self-confidence.

#### Analysis of Potential Confounders

In exploring potential confounders, comparison of PPH and NR mean knowledge scores by provider gender across the intervention and control groups combined showed that there were significant differences along gender lines in the pre-test for both PPH and NR; however, there were no differences between men and women for either post-test (Table 5). Mean pre-test knowledge scores for men across both intervention and control groups were statistically significantly higher for both PPH and NR knowledge (53.9, SD=13.8 and 53.6, SD=15.9, respectively) compared with women's scores (43.4, SD=15.6 and 40.3, SD=16.9, respectively) (*P*=.008 for PPH differences between men and women and *P*=.003 for NR differences between men and women). In contrast, men in the post-test had similar mean scores compared with those of the women on both tests. The mean increase in PPH knowledge from pre- to post-test among women was statistically significantly larger compared with the increase among men (13.8, SD=17.3 vs. 5.7, SD=11.8, respectively; *P*=.046). Similarly, the mean increase in NR knowledge among women was statistically significantly larger compared with the increase among men (12.0, SD=19.0 vs. 0.8, SD=15.7, respectively; *P*=.02).

**TABLE 5.** Within- and Between-Subject Differences in Knowledge Scores Pre- and Post-Intervention (Among Intervention and Control Groups Combined), Analyzed by Gender and Smartphone Experience

	Men (n=25)	Women (n=37)	Difference Between Men and Women	95% CI	*P* Value	Cohen's d	Never Used Smartphone (n=39)	Experienced Smartphone (n=23)	Difference Between Smartphone Experience	95% CI	*P* Value	Cohen's d
**PPH knowledge scores (out of 100)**												
Pre, mean (SD)	53.9 (13.8)	43.4 (15.6)	10.5	(2.8, 18.3)	.008	0.72	43.0 (14.5)	55.6 (14.6)	12.6	(5.0, 20.3)	.002	.87
Post, mean (SD)	59.6 (15.3)	57.2 (18.9)	2.5	(−6.6, 11.6)	.588		53.8 (17.2)	65.6 (15.7)	11.8	(3.0, 20.5)	.009	.72
Pre-post difference, mean (SD)	5.7 (11.8)	13.8 (17.3)	8.1	(0.1, 16.0)	.046	0.56	10.9 (17.8)	10.0 (11.6)	0.85	(−7.5, 9.2)	.84	
95% CI for pre-post difference	(0.9, 10.6)	(8.0, 19.5)					(5.1, 16.6)	(5.0, 15.0)				
*P* value for pre-post difference	.02	<.001					.001	<.001				
Cohen's d for pre-post difference	0.37	0.80					0.69	0.66				
**NR knowledge scores (out of 100)**												
Pre, mean (SD)	53.6 (15.9)	40.3 (16.9)	13.3	(4.8, 21.9)	.003	0.81	43.9 (17.5)	48.7 (18.0)	4.7	(−4.6, 14.0)	.31	
Post, mean (SD)	54.4 (14.8)	52.3 (18.3)	2.1	(−6.7, 10.9)	.634		51.5 (17.8)	55.9 (15.0)	4.3	(−4.5, 13.2)	.33	
Pre-post difference, mean (SD)	0.8 (15.7)	12.0 (19.0)	11.2	(2.1, 20.4)	.02	0.66	7.6 (19.4)	7.2 (17.0)	.40	(−9.4, 10.2)	.94	
95% CI for pre-post difference	(−5.7, 7.2)	(5.6, 18.3)					(1.3, 13.9)	(−0.1, 14.6)				
*P* value for pre-post difference	.81	<.001					.02	.05				
Cohen's d for pre-post difference		0.68					0.43	0.44				

Abbreviations: CI, confidence interval; NR, neonatal resuscitation; PPH, postpartum hemorrhage; SD, standard deviation.

Analysis of test scores by previous smartphone experience showed significant differences for mean PPH scores on both pre- and post-tests across both intervention and control groups combined, wherein people with previous smartphone experience scored statistically significantly higher on the PPH pre- and post-tests (*P*<.05) (Table 5). Although providers who had smartphone experience scored slightly higher on the NR pre- and post-tests, there was no significant difference. Similarly, there was no significant difference in mean change in PPH and NR knowledge from pre- to post-test among those experienced with smartphones and those who had never used them.

ANOVA tests were employed to examine differences in test score results by health professional cadre using a breakdown of all participants across both intervention and control groups together into 3 cadres (nurses, midwives, and MDs) (Table 6). The ANOVAs indicated significant group mean differences for the 3 cadres only for the NR pre-test. For both NR and PPH pre-tests, MDs had the highest scores, followed by midwives and then by nurses. In both post-tests, nurses scored higher than midwives, with MDs scoring the highest. This difference reflects positively on the internal validity of the measurement instruments to distinguish differences between cadres.

**TABLE 6.** Knowledge Scores Pre- and Post-Intervention (Among Intervention and Control Groups Combined), Analyzed by Health Worker Cadre

	Scores, Mean (SD)				F	*P* Value	*P* Value for Paired Comparisons^a^		
	Nurses (n=36)	Midwives (n=10)	MDs (n=16)	All Cadres (N=62)	Nurses–Midwives	Nurses–MDs	Midwives–MDs
**Pre-tests**									
PPH	44.4 (16.8)	48.8 (14.1)	54.1 (12.3)	47.6 (15.7)	2.197	.12	—	—	—
NR	41.1 (18.5)	47.5 (11.8)	54.9 (15.5)	45.7 (17.7)	3.747	.03	—	.02	—
**Post-tests**									
PPH	56.9 (17.6)	53.2 (17.5)	64.1 (16.7)	58.2 (17.5)	1.428	.25	—	—	—
NR	53.4 (15.1)	42.8 (21.2)	59.0 (15.6)	53.2 (16.9)	3.046	.055	—	—	.04

Abbreviations: MD, medical doctor; NR, neonatal resuscitation; PPH, postpartum hemorrhage; SD, standard deviation.

a Significant for Tukey's HSD (honestly significant test).

#### Patient Outcomes

In terms of patient outcome data, birth and maternal mortality figures were collected by register review of hand-entered data in the 8 study facilities. These data were triangulated with data collected by mobile phones using the ODK application and the monthly HIS reporting. ODK-generated data corresponded well with hand-collected register data; however, the HIS data differed from the ODK and register data. Patient adverse events were too few to compare statistically, given the small number of facilities and months in the study period, as well as infrequent occurrence of maternal death. Obstetric complication and BEmONC signal function execution data collection was piloted with the ODK, but is not collected systematically by the facility registers and is not captured by the HIS reporting; therefore, these data were not possible to triangulate. Since the ODK was only introduced with the intervention (in May 2017), we were not able to compare ODK data pre- and post-test.

### Qualitative Data

Responses for SDA users were coded to 8 out of 14 domains:

- Knowledge
- Skills
- Belief about capabilities
- Reinforcement
- Intentions
- Emotion
- Memory/attention/decision processes
- Environmental context and resources

Key stakeholder responses were mapped to these same 8 domains, plus 2 additional domains (social/professional role and identity, beliefs about consequences), since the discussion took into account the broader issues and context of CE in the DRC. The scope of analysis was limited to the above domains.

#### Interviews With SDA Users

The 8 interviewees discussed how use of the SDA was feasible and acceptable. They perceived a positive effect on their knowledge, skills, belief about capabilities/confidence, intentions, memory/attention/decision processes, and emotion (Table 7). Many noted that it helped to “hear and see [the information] at the same time.” One respondent said, “We did things blindly before with what we learned in school and it wasn't enough.”

**TABLE 7.** Qualitative Interview Results With SDA Users in Intervention Facilities (n=8)

Domains	Illustrative Quotes
Knowledge	“When we hear and see [the information] at the same time it teaches a lot.” “We did things blindly before with what we learned in school and it wasn't enough.”
Skills	“Training gave us the skills to use the app.”
Beliefs about capabilities	“We see that misoprostol is effective [for PPH].” “Had many PPH deaths before … PPH resolves if you use what is in the video.” “Now we see less fever in children after NR when we give antibiotics.” “With info in the video for NR, you see the newborn coming back. It's really encouraging.”
Reinforcement	“We watched videos about every 3 days during free time at maternity [ward] alone or with maternity team. Also used during a case of manual removal of placenta and MVA.” “One can re-watch the video as many times as one wants.”
Intentions	“It changed our old habits” “Now we take vital signs and use the partogram during delivery.” “Before for AMSTL we put the baby off to the side; now we put baby skin-to-skin and encourage breastfeeding.” “We aspirated all babies; now we only aspirate when we need to.” “Before we held the baby upside down after delivery and gave mouth-to-mouth brutally if needed; now we use the Ambu bag, which gives a good result. We learned that we must position the baby and the mask in order to do NR.” “Before for respiratory distress we did mouth to mouth and gave hydrocortisone IM, no antibiotics, and saw high rate of fever. Now, we give antibiotics, and we see less fever.” “Before we didn't do uterine massage or use misoprostol or IV fluids for PPH management; now we use massage, misoprostol, and IV fluids … with good results.” “Now we do uterine massage [with PPH] and use a urinary catheter, and we see the uterus contracts.” “Before we pushed the uterus down during 3rd stage; now we support the uterus and use controlled traction on the cord.” “Now with premature rupture of membranes we give antibiotics.”
Memory, attention, and decision processes	“mLearning with the app is good, the learner sees the information, hears it and then can do it themselves. It helps participants to remember the visual images or auditory information.”
Environmental context and resources	“Only 2 of us were trained back in 2012 but need others to be trained, and we need formative supervision more often.” “Need uniforms, tops, shoes, eye protective equipment, aprons, soap. We work in our own clothes and shoes, we risk to contaminate our children.” “We earn nothing–12,700 Francs per month. Put yourself in our place. We work hard for nothing.”
Emotion	“It's amusing and relaxing. It's good for educating adults. There is variety.” “App should be made more widely available–in pediatrics and the operating room.” “Animated graphics were interesting. Other trainings, they talk and talk.” “We are very happy with the intervention. It's very encouraging.”

Abbreviations: AMSTL, active management of the third stage of labor; IM, intramuscular; IV, intravenous; MVA, manual vacuum aspiration; PPH, postpartum hemorrhage; NR, neonatal resuscitation; SDA, Safe Delivery App.

Respondents reported changes in their intentions, belief about capabilities, and memory/attention/decision processes, which led them to change their management of BEmONC, including now taking vital signs, using uterine massage and bimanual compression in PPH, using controlled cord traction during the third stage of labor, giving intravenous fluids and misoprostol for PPH, and using the partogram. One respondent said, “It changed our old habits.” The respondents discussed observed changes in patient outcomes as a result of using the SDA and how this reinforced their new practices, such as: “There are no more deaths from PPH now.” “All the children are saved with using the Ambu bag for NR.” “Now we see less fever in children after NR when we give antibiotics.” “PPH resolves if you use what is in the video.” “With info in the video for NR, you see the newborn coming back. It's really encouraging.”

In terms of emotion, one participant said: “[The videos] are amusing and relaxing. It's good for educating adults. There is variety.” Another said: “The animated graphics were interesting. Other trainings, they talk and talk.” The interviewees stated that they consulted the app frequently (reinforcement), both as a learning tool and in various obstetric and neonatal emergencies as a job aid. The app topic most consulted by the respondents was PPH management (n=8). Participants preferred watching the animated videos, as compared with the written app features, and many of the providers interviewed had not consulted the other features in the app. After the PPH video, the other videos most frequently watched by the respondents were on topics related to active management of the third stage of labor (n=5), NR (n=4), eclampsia (n=2), newborn care (n=2), septicemia (n=1), manual vacuum aspiration (n=1), manual extraction of the placenta (n=1), and prolonged labor (n=1).

The app topic most consulted by the respondents was PPH management.

In terms of barriers to implementation of the SDA and BEmONC guidelines, participants cited environmental context and resources, particularly the poor practice environment, lack of consistent medications, equipment, electricity, and poor salary. One respondent said, “Availability of material would help us to manage better: uniforms, tops, shoes, eye protective equipment, aprons, soap. We work in our own clothes and shoes, we risk to contaminate our children.” Another said, “We have needs for certain materials to carry out work properly: long gloves and lights [maternity ward has no power].” “We earn nothing. Put yourself in our place. We work hard for nothing.”

#### Interviews With Key Stakeholders

Data from semistructured interviews with 10 key stakeholders mapped to all of the same domains noted with the SDA users, plus an additional 2 domains (social/professional role and identity and beliefs about consequences) (Table 8).^30^^–^^31^ Respondents supported the feasibility and acceptability of mLearning and the potential for it to have an impact on maternal and neonatal mortality. Many respondents noted that health workers often have no access to CE and that the same people are sometimes selected many times for training. One respondent reported, “There is a poor distribution of opportunity to get CE. There is limited training for hard-to-reach areas and for lower professional cadres.” Another interviewee noted, “eLearning can train more people at lower cost.”

**TABLE 8.** Qualitative Interview Results With Key Stakeholders (n=10)

Domains	Illustrative Quotes
Knowledge	“Trainings are theoretical via lectures. They teach too many things at the same time and they rush through the material. Learners have trouble prioritizing and leave with confusion; meanwhile the essential notions aren't mastered and they aren't able to apply the knowledge to a case. Providers have been trained before but it's as if they have never been trained. It would be better to have more practical training.” “CE should incorporate adult learning principles, and it should be continuous/regular.” “Hospital/workers are often 'subjected' to trainings that they haven't planned … should be included in planning in response to priority gaps/needs.” “Must present things that are relevant to what they [health workers] do–where they get practical info and can see the gestures.” “SDA renders learning operational.”
Skills	“There is a weak development of competence of personnel.” “Care has become mechanized and based on memorized protocols, so they [health workers] have a difficult time analyzing situations.” “Providers were able to put into practice new things learned [from the SDA] such as using the side of the hand for manual extraction [of the placenta] and bimanual compression for PPH.” “Should be conducted in real work conditions to combat the gap between what one knows and what one does.” “mLearning also teaches people how to use technology.”
Social/professional role and identity	“[Health workers] use what they have to treat patients, but they are not going to look something up or get additional info.” “Must create a way for people to share information. Maybe form a club (to discuss with an animator/trainer). If the program is personalized, even better–get points, get certificate [must be linked to the employer].”
Beliefs about capabilities	“Imposing CE is only of limited value, when the boss isn't there … they won't do it.” “Inspiring them to the benefits of CE would be more motivating then sanctions.”
Beliefs about consequences	“No accountability related to malpractice.” “CE regulations/requirements are where the country needs to go to make a change.” “[Employers or] Ministry should have tracking capability related to CE to be able to identify persons in need of training and needed training.” “Must assure career path for health workers based on regular evaluation.” “Must lead to a change in employment status/have a concrete change.”
Reinforcement	“mLearning is interesting to reinforce learning since the tool is available at all times and the provider can view the information many times.” “Ideal if someone [trainer] follows the process to enrich the application [give retro-information and ensure that needs are covered in the app], and to answer questions.” “SDA should be made available in all health zones with post-training supervision … would decrease maternal and neonatal mortality.”
Intentions	“For remote settings, eLearning is interesting–self-directed learning, and should measure if they acquire competencies/can be certified, and put in place a system for them to be encouraged to do this.”
Memory, attention, and decision processes	“The images allow people to learn with more stimulation and attention. The visual memory can fix the memory of the information for longer.” “When time passes after a training, you forget.” “If people are motivated they may retain info better.”
Environmental context and resources	“Better working conditions [salaries, supervision, environment] would push people.” “Poor distribution of opportunity to get CE training. Limited training for hard-to-reach areas and for lower health worker cadres (A3, A2).” “For CE, programs/partners decide subject, may repeat subjects already covered, same people always go to the trainings and are missing from work.” “No clear CE policy. Who chooses the subjects? And must be defined who participates and what one gets from it. Needs a clear policy, training should be programmed and budgeted … so that employers recognize the training and has it planned/budgeted for their facility.” “eLearning can train more people at lower cost.”
Emotion	“Seeing the images is more interesting than just talking theoretically.” “Approaches must change from written info to interactive self-learning options.” “New technology fascinates/attracts people and they want to try it; it responds to a need.”

Abbreviations: CE, continuing education; PPH, postpartum hemorrhage; SDA, Safe Delivery App.

Respondents noted that current trainings are often too theoretical, are not necessarily relevant to the daily work of health workers, are not of interest, and don't do a good job of enhancing knowledge, skills, or changing belief about capabilities or intentions to change behavior. One respondent said, “Generally, there is a weak development of competence of personnel with current continuing education.” Another said, “We must change training approaches to those that facilitate learning. Approaches aren't adapted to the current era, using written modules and lectures. People don't read the modules.” One service leader said, “Trainings are too theoretical via lectures; the essential notions aren't mastered and trainees aren't able to apply the knowledge to a case.”

To combat infrequent access to training or self-directed learning opportunities, mLearning was noted to provide the opportunity for reinforcement of learning and to be more interesting (emotion, memory/attention/decision processes): “New technology fascinates people and they want to try it. It responds to a need or desire for learning.” “New technology should be encouraged, especially for remote areas.” “Audiovisual makes it more interesting and one can experience it alone or in a group. Approaches must change from written info to interactive self-learning options.”

In terms of barriers to mLearning or CE, responses centered on the domain of beliefs about consequences, and noted the lack of incentives or requirements for CE, amidst the general lack of national and regional planning and tracking capacity for CE. One interviewee noted, “There is no link between CE and career progression.” Others said, “CE must meet a need, fill a gap, and lead to a change in employment status or a concrete change.” “We need an accreditation system for CE, so that people have to take CE with a systematic plan of courses required for different fields.” One respondent noted, “mLearning should be linked with post-training monitoring and supervision and it should be connected to performance contracts.” Another said, “We must encourage health workers to do better.” Interviewees also noted that many deficiencies centered on the domain of environmental context and resources, highlighting the contextual gaps that result in poor care such as lack of accountability for poor practice, insufficient remuneration for health workers, lack of drugs, equipment, and supervision. One respondent noted the differences between what people are taught and the reality of the environment: “challenges are linked to logistics: electricity, equipment.”

### Triangulation of Quantitative and Qualitative Data

Qualitative interviews identified many positive benefits to the use of the SDA and mLearning in the DRC context, particularly in terms of making evidence-based, up-to-date global BEmONC guidelines available to health workers via an exciting mLearning app. Access to information was noted to be especially critical to those who are often devoid of learning opportunities such as those in remote areas and lower-level cadres. However, interviewees noted that even for those who have been trained in the past, the SDA and mLearning offer the opportunity to learn important knowledge and skills and change behavior via the use of a more modern, more captivating approach that appeals to all health personnel. This reinforces our quantitative results of significantly increased knowledge and self-confidence scores (which directly mirror the 2 domains of knowledge and beliefs about capabilities) across all 3 health professional cadres (MDs, nurses, midwives) after 3 months of SDA use. The increases were unaffected by previous smartphone use, reinforcing that mLearning can be used to train any health worker.

Qualitative responses further elucidated barriers to mLearning and CE that are well known to key stakeholders in the DRC, such as environmental and contextual barriers and lack of resources (Table 8). The policy context, including lack of accountability and incentivizing measures (beliefs about consequences) and gaps in professional identity, mutually reinforce the environmental context and resource gaps, contributing substantially to poor quality of care by health care providers and high mortality indicators for mothers and newborns.

## DISCUSSION

Learning via the SDA was feasible and acceptable for health workers in the context of the DRC. mLearning, more broadly, was assessed by our sample of key stakeholders in the DRC to be feasible, acceptable, and a potential solution to health workers' problems of accessing up-to-date learning resources in hard-to reach settings. A pilot trial with the French-language version of the SDA in the DRC led to a significant increase in health care workers' knowledge scores for PPH and NR management and in BEmONC self-confidence scores in intervention as compared with control participants, irrespective of previous smartphone use or professional cadre of the health worker.

Learning via the Safe Delivery App was feasible and acceptable for health workers in the DRC.

This study supported findings by Lund et al.^14^ regarding the significant effect of the use of the SDA with skilled birth attendants in Ethiopia in terms of significantly increased knowledge and skill scores of health workers on neonatal resuscitation and a non-significant 24% reduction in perinatal mortality. The trial reported in this article did not test BEmONC skill scores given the smaller nature of the feasibility study. Similarly, this trial was unable to determine the impact of the SDA on patient outcomes with sufficient power, given the small sample size and short duration of the study combined with relatively infrequent occurrence of maternal death. However, the research team did assess the feasibility of study procedures for a future larger well-powered study in the DRC.

Despite being unable to demonstrate 2 key needs for mHealth trials in LMICs identified in systematic reviews,^21^^–^^23^ namely, trials with patient outcomes as a primary outcome and longer-term trials, this study did assess the feasibility, acceptability, and potential efficacy of using the SDA to improve the quality of BEmONC in the largest francophone African country. It also proposed a potential means of addressing the challenge of inadequate access to up-to-date evidence-based training and reference materials for health workers in hard-to-reach areas and for health worker cadres that often miss out on training opportunities in the DRC. The advantages of the SDA are that it is self-explanatory, available in many languages, is open source and free for download, and, once installed on the mobile device, does not need network coverage to function.

The Safe Delivery App is open source, free, and available in many languages.

Conventional training of skilled birth attendants in BEmONC has proved effective to improve health care outcomes.^11^^,^^32^^,^^33^ However, health care workers in hard-to-reach settings are often not able to participate in such trainings and are unable to access other learning resources.^10^^,^^17^^,^^18^ Systematic reviews on mHealth show that mobile phone applications are increasingly being used in LMICs to disseminate information to health care workers.^34^ Other pilot studies have shown related eLearning strategies to be potentially as effective as traditional training strategies.^20^ These findings also support those of other studies that the use of electronic tools is perceived as an opportunity for improving health worker quality of care with effects on health care workers' motivation,^35^ self-efficacy,^36^ and enthusiasm.^37^ The Theoretical Domains Framework proposes domains of influence or constructs for health worker behavior change that mirror these concepts, with domains such as belief about capabilities, intentions, and emotion as being critical determinants of behavior change.^30^^–^^31^

Lessons from this feasibility study to improve future study procedures suggest that future trials of mLearning in the DRC would benefit from an additional means of data collection for mortality and other critical BEmONC data, such as through the use of the ODK data collection instrument designed for this study or dedicated data collection staff on-site. Researchers found that data collected by hand-review of health facility registers and ODK data collected daily via mobile phone were comparable, but differed from monthly HIS reporting. Although not the primary question in our research, interview data revealed possible explanations for discrepancies in data collected from these different sources including under-reporting of mortality in the HIS by health workers for a variety of reasons such as incomplete compilation of HIS data using only certain registers (maternity) rather than all related hospital registers where maternal or newborn deaths would be recorded (such as emergency, gynecology, and pediatric services), or omission in reporting deaths given that HIS data are only collated on a monthly basis and recording may be done retrospectively. Under-reporting of adverse outcomes was mentioned to be deliberate on occasion, due to fear of punishment by health authorities, or financially motivated. Such factors would clearly complicate the measurement of patient outcomes in an eventual follow-up study using HIS data alone in the DRC unless certain remedial measures are taken to improve mortality data quality.

Future work could also benefit from use of the recently updated version of the SDA, which incorporates additional features to measure learning and to motivate the user through game-like features, where learners must gain a certain number of points to move to the next learning level and are certified once they achieve the top score. These features might exponentially increase the benefits of the SDA intervention by providing incentives and rewards, and promoting motivation and self-empowerment, thereby touching on domains such as emotion, reinforcement, and belief about capabilities. These features would rely on Internet connectivity and bandwidth, which remain out of reach for individuals in many contexts due to poor connections or lack of financial access, despite rapid increases of the use of wireless communication in many developing countries.^34^

The recently updated version of the Safe Delivery App incorporates additional features to measure learning and motivate the user, such as game-like features.

The implications of this study are that the SDA and other mLearning interventions likely increase the ability of health workers to provide improved quality of care during obstetric and neonatal emergencies as well as improved routine obstetric and newborn care. Evolving policies for continuing education in the DRC and similar contexts should consider the integration of mLearning as an approach for training and as a job aid for EmONC in order to reduce maternal and newborn mortality, as well as considering the integration of mLearning tools for other priority and emergent health problems. Challenges to implementation of quality EmONC care, posed by gaps in the environmental context and resources, as well as the regulatory and accountability environment, must be considered and addressed alongside other programmatic measures such as quality improvement initiatives to target health system weaknesses.

### Limitations

The small sample size of this study limited findings by reducing the power of the study. In terms of design limitations, 8 facilities were randomized in this study, rather than individuals, to avoid contamination of the intervention group to the control group,^14^ and researchers did note disparities between the intervention and control groups in the gender, professional cadre composition, and previous experience of the health workers. Additionally, blinding of intervention and control clusters was impossible owing to the nature of the intervention, which increased the risk for information bias. It is possible, however, that some control participants accessed the SDA in intervention facilities, since the facilities were all in relatively close geographic approximation. Other limitations included that the study team was unable to consistently track SDA use during the study, given that this capability was not completely developed at the time of intervention. Access to such data would enrich results and analysis of the association between SDA use and changes in measures in future research.

## CONCLUSION

The SDA and mLearning was found, through both qualitative and quantitative methods, to be feasible and acceptable to health workers and to key stakeholders in the DRC, the largest francophone African country. SDA use was associated with increased health worker knowledge on PPH and NR management 3 months after introduction and increased health worker self-confidence overall in the management of obstetric and newborn emergencies. These results contribute to the growing body of knowledge on mHealth in low-income countries where the quality of care is challenged by lack of continuing education programs.

## Supplementary Material

GHSP-D-18-00275-Fr-version.pdf

## Notes

**mLearning en République Démocratique du Congo : Un Essai Randomisé Pilote par Grappe Utilisant des Méthodes Mixtes à l'Aide de l'Application « Safe Delivery »**

Les connaissances des professionnels de santé et leur confiance en soi dans les soins obstétricaux et néonatals d'urgence de base (SONU-B) se sont significativement améliorées trois mois après l'introduction de l'application « Safe Delivery » dans les établissements de santé d'intervention par rapport aux établissements témoins.

**RÉSUMÉ**

**Contexte :** Le manque de qualité des soins à l'accouchement a été largement documenté comme cause majeure indirecte des décès maternels dans les établissements de santé au niveau mondial. Bien que l'apprentissage des professionnels de santé via des téléphones mobiles (mLearning) augmente rapidement, il existe peu de preuves de l'efficacité du mLearning. Cette étude visait à déterminer la faisabilité, l'acceptabilité et les effets potentiels de l'application « Safe Delivery » (SDA) sur les pratiques des professionnels de santé en matière de soins obstétricaux et néonatals d'urgence de base (SONU-B) en République Démocratique du Congo (RDC). Le « Cadre des Domaines Théoriques » a été utilisé pour guider cette recherche.

**Méthodes :** Huit établissements de santé de SONU-B situés dans le centre-est de la RDC ont été sélectionnés de façon randomisée pour recevoir une intervention de mLearning ou être établissements témoins. Les professionnels de santé en charge de la santé maternelle et néonatale dans les établissements d'intervention (n = 64) ont été formés à l'utilisation des smartphones et à la version française de la SDA. La SDA est une ressource de formation en SONU-B basée sur des preuves empiriques. Il s'agit d'une application visuelle qui utilise à la fois des vidéos et des instructions de prise en charge clinique développées par la Maternity Foundation et les universités de Copenhague et du Danemark du Sud. Les connaissances sur l'hémorragie du post-partum (HPP) et la réanimation néonatale (RN) ainsi que la confiance en soi pour l'exécution de 12 procédures SONU ont été évaluées au départ et trois mois après l'intervention de mLearning. Dix-huit entretiens qualitatifs ont été menés avec les utilisateurs de l'application SDA et les principales parties prenantes afin d'évaluer la faisabilité et l'acceptabilité du mLearning, ainsi que l'utilisation de la SDA. La mortalité maternelle a été comparée dans les établissements d'intervention et les établissements témoins à l'aide d'une application de données Open Data Kit (ODK), qui utilise également les smartphones. Des smartphones avec les applications et ODK ont été confiés aux établissements d'intervention pour la période de l'étude, tandis que les établissements du groupe témoin n'ont reçu que des smartphones avec ODK.

**Résultats :** L'analyse a porté sur 62 professionnels de santé. Les scores de connaissances sur l'hémorragie du post-partum et la réanimation néonatale ont augmenté de manière significative par rapport aux valeurs initiales chez les participants à l'intervention par rapport aux participants témoins trois mois après l'intervention. En effet, la différence moyenne pour les connaissances sur l'HPP est de 17,4 sur 100 (intervalle de confiance [IC] 95% = 10,7 à 24,0) et de 19,4 pour les connaissances sur RN (IC 95% = 11,4 à 27,4). De plus, les scores de confiance en soi pour 12 procédures SONU ont également significativement augmenté (différence moyenne 4,2 sur 48, IC 95% = 0,7 à 7,7). Les améliorations n'ont pas été affectées par le profil des professionnels de santé ni l'utilisation antérieure du smartphone. Des entretiens qualitatifs ont confirmé la faisabilité et l'acceptabilité de la SDA et du mLearning, ainsi que le potentiel impact de ces approches sur la mortalité maternelle et néonatale en RDC.

**Conclusion :** L'utilisation de l'Application « Safe Delivery » a permis d'accroitre les connaissances des professionnels de santé et leur confiance en soi dans la prise en charge des urgences obstétricales et néonatales après trois mois. La SDA et le mLearning ont été jugés faisables et acceptables pour les professionnels de santé et les principales parties prenantes en RDC.

## Notes

**Peer Reviewed**

**Funding**: IMA World Health.

© Bolan et al. This is an open-access article distributed under the terms of the Creative Commons Attribution 4.0 International License (CC BY 4.0), which permits unrestricted use, distribution, and reproduction in any medium, provided the original author and source are properly cited. To view a copy of the license, visit http://creativecommons.org/licenses/by/4.0/. When linking to this article, please use the following permanent link: https://doi.org/10.9745/GHSP-D-18-00275
