# Supplementary material for: mLearning in the Democratic Republic of the Congo: A Mixed-Methods Feasibility and Pilot Cluster Randomized Trial Using the Safe Delivery App
Source: Glob Health Sci Pract. 2018 Dec 27;6(4):693–710. doi: 10.9745/GHSP-D-18-00275 (PMC6370362; doi:10.9745/GHSP-D-18-00275)
Supplement: GHSP-D-18-00275-Fr-version.pdf [file GHSP-D-18-00275-Fr-version.pdf]

GLOBAL HEALTH: SCIENCE AND PRACTICE
Dedicated to what works in global health programs
OPEN ACCESS

ARTICLE ORIGINAL

mLearning en République Démocratique du Congo: Un Essai Randomisé Pilote par Grappe Utilisant des Méthodes Mixtes à l'Aide de l'Application « Safe Delivery »

Nancy E. Bolan,[a] Larry Sthreshley,[b] Bernard Ngoy,[b] Faustin Ledy,[b] Mano Ntayingi,[b] Davis Makasy,[b] Marie-Claude Mbuyi,[c] Gisele Lowa,[b] Lynne Nemeth,[a] Susan Newman[a]

Les connaissances des professionnels de santé et leur confiance en soi dans les soins obstétricaux et néonatals d'urgence de base (SONU-B) se sont significativement améliorées trois mois après l'introduction de l'application « Safe Delivery » dans les établissements de santé d'intervention par rapport aux établissements témoins.

RÉSUMÉ

Contexte: Le manque de qualité des soins à l'accouchement a été largement documenté comme cause majeure indirecte des décès maternels dans les établissements de santé au niveau mondial. Bien que l'apprentissage des professionnels de santé via des téléphones mobiles (mLearning) augmente rapidement, il existe peu de preuves de l'efficacité du mLearning. Cette étude visait à déterminer la faisabilité, l'acceptabilité et les effets potentiels de l'application « Safe Delivery » (SDA) sur les pratiques des professionnels de santé en matière de soins obstétricaux et néonatals d'urgence de base (SONU-B) en République Démocratique du Congo (RDC). Le « Cadre des Domaines Théoriques » a été utilisé pour guider cette recherche.

Méthodes: Huit établissements de santé de SONU-B situés dans le centre-est de la RDC ont été sélectionnés de façon randomisée pour recevoir une intervention de mLearning ou être établissements témoins. Les professionnels de santé en charge de la santé maternelle et néonatale dans les établissements d'intervention (n = 64) ont été formés à l'utilisation des smartphones et à la version française de la SDA. La SDA est une ressource de formation en SONU-B basée sur des preuves empiriques. Il s'agit d'une application visuelle qui utilise à la fois des vidéos et des instructions de prise en charge clinique développées par la Maternity Foundation et les universités de Copenhague et du Danemark du Sud. Les connaissances sur l'hémorragie du post-partum (HPP) et la réanimation néonatale (RN) ainsi que la confiance en soi pour l'exécution de 12 procédures SONU ont été évaluées au départ et trois mois après l'intervention de mLearning. Dix-huit entretiens qualitatifs ont été menés avec les utilisateurs de l'application SDA et les principales parties prenantes afin d'évaluer la faisabilité et l'acceptabilité du mLearning, ainsi que l'utilisation de la SDA. La mortalité maternelle a été comparée dans les établissements d'intervention et les établissements témoins à l'aide d'une application de données Open Data Kit (ODK), qui utilise également les smartphones. Des smartphones avec les applications et ODK ont été confiés aux établissements d'intervention pour la période de l'étude, tandis que les établissements du groupe témoin n'ont reçu que des smartphones avec ODK.

Résultats: L'analyse a porté sur 62 professionnels de santé. Les scores de connaissances sur l'hémorragie du post-partum et la réanimation néonatale ont augmenté de manière significative par rapport aux valeurs initiales chez les participants à l'intervention par rapport aux participants témoins trois mois après l'intervention. En effet, la différence moyenne pour les connaissances sur l'HPP est de 17,4 sur 100 (intervalle de confiance [IC] 95% = 10,7 à 24,0) et de 19,4 pour les connaissances sur RN (IC 95% = 11,4 à 27,4). De plus, les scores de confiance en soi pour 12 procédures SONU ont également significativement augmenté (différence moyenne 4,2 sur 48, IC 95% = 0,7 à 7,7). Les améliorations n'ont pas été affectées par le profil des professionnels de santé ni l'utilisation antérieure du smartphone. Des entretiens qualitatifs ont confirmé la faisabilité et l'acceptabilité de la SDA et du mLearning, ainsi que le potentiel impact de ces approches sur la mortalité maternelle et néonatale en RDC.

Conclusion: L'utilisation de l'Application « Safe Delivery » a permis d'accroitre les connaissances des professionnels de santé et leur confiance en soi dans la prise en charge des urgences obstétricales et néonatales après trois mois. La SDA et le mLearning ont été jugés faisables et acceptables pour les professionnels de santé et les principales parties prenantes en RDC.

[a] College of Nursing, Medical University of South Carolina, Charleston, SC, USA.
[b] IMA World Health, Kinshasa, République Démocratique du Congo.
[c] Pathfinder International, Kinshasa, République Démocratique du Congo.
Correspondance à Nancy E. Bolan (Nancy.e.Bolan@uliege.be).

INTRODUCTION

Les prestations cliniques des professionnels de santé sont souvent insuffisantes dans les pays à revenu faible ou intermédiaire.[1] Le manque de qualité

des services en matière d'accouchement et de soins obstétricaux et néonatals d'urgence (SONU) a été largement documentés comme l'une des principales causes de mortalité chez les mères et les nouveau-nés dans les établissements de santé au niveau mondial.[2] Environ 830 femmes meurent chaque jour dans le monde de causes évitables liées à la grossesse et à l'accouchement[3] et près de deux millions de nouveau-nés meurent chaque année au cours de la première semaine de vie suite à des complications au moment de l'accouchement.[4] 99% de tous les décès maternels surviennent dans des pays en développement (302000); l'Afrique subsaharienne compte pour environ 66% des décès maternels (201000) survenant dans le monde entier.[5]

La République Démocratique du Congo (RDC), le plus grand pays d'Afrique subsaharienne, a l'un des taux de mortalité maternelle les plus élevés d'Afrique (846 décès maternels pour 100000 naissances vivantes).[6] Le risque de décès maternel d'une femme au cours de sa vie (probabilité qu'une femme de 15 ans décède d'une cause maternelle au cours de sa vie), est estimé à 1 sur 24 en RDC, contre 1 sur 3300 dans les pays à revenu élevé.[5] Le taux de décès néonataux (décès survenant avant 28 jours de vie) est estimées à 29 pour 1000 naissances vivantes en RDC.[4] À l'échelle mondiale, les disparités en matière de santé maternelle et infantile reflètent largement les inégalités d'accès à des services de santé de qualité.[3]

Les déficits en connaissances et compétences des professionnels de santé sont liés aux faibles résultats chez les patients dans les contextes à ressources limitées.[7–9] Les prestataires de soins maternels présentent un faible niveau de connaissances sur les SONU, malgré des années d'expérience comme prestataire, et ne possèdent pas les compétences requises pour la prise en charge de l'HPP.[2,10–12] L'HPP est la principale cause de mortalité maternelle dans le monde,[13] et sa prise en charge est l'une des sept « fonctions clés » des SONU de base (SONU B). Ces fonctions représentent des interventions médicales clés devant être effectuées par tous les personnels qualifiés à l'accouchement. Il existe en la matière un écart important entre les professionnels de santé des établissements de santé périphériques, qui ne sont pas suffisamment formés pour prendre en charge les urgences obstétricales, et ceux des établissements urbains.[14] En outre, dans les contextes à faible volume d'accouchements, les complications obstétricales ne sont pas assez nombreuses pour permettre aux prestataires d'acquérir une expérience solide en SONU.[15]

Une stratégie de base pour modifier le comportement des professionnels de santé et renforcer la qualité des prestations cliniques consiste à promouvoir la formation continue (FC) ou le développement professionnel continu.[16] Cependant, pour de nombreux professionnels de santé, l'accès à des opportunités de formation actualisées et pertinentes est difficile, voire impossible, en particulier dans les zones à accès difficile ou périphériques, où la mortalité maternelle et néonatale est généralement la plus élevée.[10,17,18] Cependant, la disponibilité et l'utilisation des téléphones mobiles ainsi que l'apprentissage via ces appareils (« mLearning ») augmentent rapidement dans les pays à revenu faible ou intermédiaire.[19] Compte tenu des coÃts et des défis logistiques liés à l'organisation des programmes de FC classiques dans les milieux périphériques, l'utilisation de téléphones mobiles et d'autres appareils électroniques mobiles semble prometteuse en tant que nouveau mécanisme pouvant permettre de fournir des informations actualisées aux prestataires les plus éloignés.[20] Cependant, parmi les études examinant le mLearning, la qualité méthodologique est souvent faible et peu d'entre elles ont évalué les effets sur les résultats pour la santé du client.[21–23]

Cette essai pilote avait pour but de déterminer la faisabilité et l'acceptabilité d'un outil de formation mLearning basé sur des preuves empiriques. Cet outil développé récemment est l'application « Safe Delivery App » (SDA). Il s'agissait de comprendre l'impact potentiel de cet outil sur la confiance et la pratique des prestataires de soins de santé travaillant dans les services de santé maternelle et néonatale en RDC. Cette recherche visait également à affiner le déroulement de l'intervention en RDC et à renforcer les procédures d'étude requises pour reproduire à terme ces méthodes sur une plus grande échelle. Cette recherche a été guidée par le Cadre des Domaines Théoriques (Theoretical Domains Framework), qui considère le changement de comportement des professionnels de santé comme un élément clé pour accroître l'utilisation des évidences dans la pratique des soins de santé.[24]

METHODES

Conception de l'étude

Cette étude pilote de faisabilité était un essai randomisé par grappe avec l'établissement de santé comme unité de randomisation. L'étude a suivi les lignes directrices CONSORT (Consolidated Standards of Reporting Trials) sur la publication des

essais pilotes et de faisabilité.[25] En utilisant une conception parallèle convergente avec des méthodes mixtes,[26] la chercheuse principale a mené des entretiens qualitatifs semi-structurés avec les utilisateurs de l'application et les principales parties prenantes.[27] En outre, les résultats sélectionnés des patients ont été comparés avant et après l'intervention. Le Comité d'Ethique de la RDC installé à l'Université Protestante du Congo (UPC) a donné son aval éthique à l'étude en avril 2017, de même que le comité de revue institutionnel de l'Université de Médecine de Caroline du Sud (MUSC)(États-Unis).

Contexte

L'étude s'est déroulée sur trois mois (avril-juillet 2017) dans deux zones de santé (Alunguli et Kindu) dans la province du Maniema, une région située au centre-est de la RDC qui dispose de peu de ressources, avec des infrastructures insuffisantes et des résultats de santé parmi les plus faibles du pays.[6] Dix établissements de santé ont constitué des sites éligibles à la randomisation par grappe du fait de leur accessibilité par véhicule et de leur désignation en tant que centres SONU-B appuyés par le projet d'Accès aux Soins de Santé Primaires (ASSP). Le projet ASSP, piloté par IMA World Health (IMA), une ONG internationale, est un projet financé par le gouvernement du Royaume-Uni (DFID) pour renforcer le système de santé et de réorganiser les soins de santé primaires. Le projet est exécuté en collaboration avec le gouvernement Congolais et des partenaires locaux et internationaux afin de revitaliser le système de santé du pays dans des zones ciblées, de lutter contre les maladies et d'améliorer les indicateurs de santé clés, en particulier ceux liés à la mortalité maternelle et infantile.[28] En tant que centres SONU, les 10 établissements de santé (1 hôpital général de référence et 4 centres de santé par zone de santé) ont reçu des produits et des équipements pour les SONU. Le personnel a également participé à des formations sur les SONU.

Randomisation

Les établissements de santé identifiés ont été stratifiés en deux catégories: hôpitaux ou centres de santé (Figure). Dans la catégorie hôpital, un établissement a été sélectionné au hasard pour l'intervention, à l'aide d'une urne remplie de papiers étiquetés, dans le groupe de deux établissements appariés. Un centre de santé a été exclu pour non-fonctionnement. Dans la catégorie des centres de santé, 3 centres ont été choisis au hasard parmi les 7 centres de santé appariés pour l'intervention et 3 comme témoins, soit un total de 4 établissements d'intervention et 4 établissements témoins (N = 8).

Participants

Les médecins, les infirmiers/ères et les sages-femmes travaillant dans les établissements de

FIGURE. CONSORT

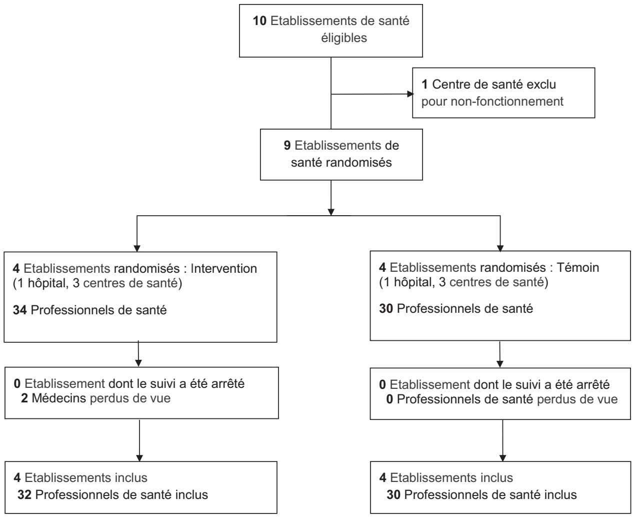

Abréviations: CONSORT, Consolidated Standards of Reporting Trials (Standards fusionnés dans la rédaction d'essais thérapeutiques)

santé sélectionnés qui gèrent les accouchements et les soins néonataux ont été invités à participer à l'étude. La population de l'étude comprenait 64 prestataires de soins dans les huit établissements de santé sélectionnés (Figure). Deux médecins du groupe d'intervention n'ont pas terminé l'étude. Ils ont passé le pré-test mais n'ont pas pu participer au tests ultérieurs en raison de problèmes de santé. Pour cette étude utilisant des méthodes mixtes, la chercheuse principale a mené des entretiens qualitatifs semi-structurés avec deux catégories de professionnels de santé pour un total cumulé de 18 entretiens. La première catégorie de professionnels de santé comprenait 10 parties prenantes clés à Kinshasa (capitale nationale) et à Kindu (capitale de la province du Maniema), et la seconde catégorie comprenait huit utilisateurs de la SDA. En ce qui concerne les parties prenantes clés, la chercheuse principale a eu recours à un « échantillonnage en boule de neige », qui repose sur les réseaux personnels des participants, pour s'adresser à d'autres personnes clés.[29] Parmi les parties prenantes clés figuraient des responsables de l'éducation, des politiques de santé, des programmes et des services de santé. Les utilisateurs de l'application ont été identifiés parmi ceux formés sur l'application depuis le début de l'étude à Kindu.[26]

Tous les participants à l'étude ont donné leur consentement verbal après avoir été informés par écrit sur les objectifs de l'étude, lors de la visite du site par l'équipe de recherche. Les informations écrites ont été approuvées par les comités d'éthique («Fiche d'information pour les participants»), assurant la confidentialité de toutes les informations obtenues au cours de l'étude et informant les participants de leur droit de se retirer de l'étude à tout moment, sans incidence sur leur situation d'emploi. Les participants à l'étude n'étaient pas masqués car l'intervention nécessitait une participation manifeste. Les établissements ont été randomisés plutôt que les individus pour éviter la diffusion des informations parmi les professionnels de santé dans le même établissement, comme la présentation de l'application et des vidéos animées par les participants du groupe d'intervention a ceux du groupe témoin.[14]

Intervention

La SDA est un outil de formation et de travail développé par la Maternity Foundation, l'Université de Copenhague et l'Université du Sud du Danemark. Elle a été conçue pour renforcer les capacités et la confiance en soi des professionnels de santé des pays à faible revenu en matière de soins obstétricaux et néonatals de base. Le contenu de l'application est basé sur les directives cliniques mondiales de SONU-B et a été validé par un groupe international d'experts en santé mondiale.[15] La SDA peut être téléchargé gratuitement pour iPhone à l'adresse suivante: https://itunes.apple.com/dk/app/safe-delivery/id985603707?mt=8et pour Android: https://play.google.com/store/apps/details?id=dk.maternity.safedelivery.

La SDA transmet des connaissances et des compétences via des vidéos animées et des instructions sur les procédures clés, y compris des informations sur les médicaments essentiels pour les SONU. Toutes les fonctions et fonctionnalités sont conçues pour les contextes à faible taux d'alphabétisation et faible revenu. Elles fonctionnent complètement hors ligne une fois téléchargées. Les 10 films d'instructions incluent les sept fonctions clés de SONU ainsi que trois procédures essentielles supplémentaires (prévention de l'infection, gestion de l'infection chez le nouveau-né et gestion active du troisième stade du travail). Dans cette étude, la version française de la SDA avait été pré-téléchargée sur les smartphones Android à Kinshasa, capitale de la RDC, en raison de la mauvaise connectivité Internet dans la région pilote (Kindu, Maniema). Un smartphone a été attribué à chaque établissement de santé de l'étude. Un instrument de collecte de données Open Data Kit (ODK), dont le contenu était conçu par les chercheurs, a également été chargé sur les smartphones afin de collecter des informations sur les statistiques des SONU et l'exécution des fonctions clés, au-delà de ce qui était normalement collecté par le système nationale d'information sanitaire ou SNIS, à travers le Système d'Information Sanitaire de District (DHIS 2). Ces informations étaient à saisir manuellement chaque jour par le personnel de l' établissement.

Le personnel des établissements sélectionnés a reçu des explications sur la nature et le but du processus. Les professionnels de santé dans les sites d'intervention ont suivi une session de formation d'une demi-journée sur l'utilisation des smartphones et des applications SDA et d'ODK. Il s'agissait d'une formation avec projection de vidéos animées et discussions communes de certaines vidéos de l'application. Dans les établissements de santé sans intervention, les prestataires dispensaient des soins standards sans l'assistance de la SDA. Cependant, une formation à la collecte de données pour l'ODK sur smartphone leur

avait été dispensée, les données étant collectées dans les établissements témoins de la même manière que dans les établissements d'intervention pendant la période de l'étude. Pour assurer la même possibilité de fournir des soins standards, la disponibilité d'un paquet minimal de médicaments et d'équipements essentiels avait été assurée par le projet ASSP dans les deux groupes d'établissements. Pendant la période d'étude de trois mois, le smartphone avec SDA était disponible pour tous les prestataires de services de maternité dans les établissements d'intervention. Des chargeurs de batterie à panneaux solaires ont été distribués aux huit installations équipées de smartphones pour garantir une capacité de charge constante. Les prestataires ont été invités à utiliser la SDA aussi souvent qu'ils le souhaitaient et à ce que le téléphone soit mis à la disposition de l'équipe de service à tout moment. Les superviseurs du Ministère de la Santé ont été chargés de visiter chaque semaine les établissements d'intervention et témoins pour rappeler aux prestataires d'utiliser l'application et/ou l'ODK.

Cadre théorique

Le Cadre des Domaines Théoriques (Theoretical Domains Framework), qui place le changement de comportement des professionnels de santé comme un élément clé pour accroître l'intégration des évidences dans la pratique des soins de santé, a été utilisé pour orienter cette recherche.[24] L'objectif initial de ce cadre était de simplifier et d'intégrer un certain nombre de théories du changement de comportement pour fournir une optique théorique permettant de visualiser les influences cognitives, affectives, sociales et environnementales sur le comportement du prestataire.[30–31] Les constructions explicatives de 33 théories du changement de comportement ont été réduites et regroupées en 14 domaines de construction théoriques, chacun constitué d'un groupe de concepts théoriques, proposés comme médiateurs potentiels du changement de comportement.[30–31] Le Cadre des Domaines Théoriques fournit une base conceptuelle utile pour évaluer les problèmes de mise en œuvre des soins fondés sur des évidences et comprendre les processus de changement de comportement des prestataires.[24] Dans cette recherche, le Cadre des Domaines Théoriques a influencé la conception des questions d'entretien visant à explorer le contenu spécifique de ces domaines en relation avec les obstacles et les facilitateurs à l'utilisation de SDA, mLearning et de la mise en œuvre de la FC en RDC. Il a également été utilisé comme cadre de codage pour l'analyse.

Résultats et Mesures

Les principaux résultats de l'essai pilote sur la SDA concernent la confiance en soi et les scores de connaissances des prestataires. Des instruments de collecte de données sur la confiance en soi et les connaissances ont été développés, testés (en anglais) et traduits en français par la Maternity Foundation à Copenhague. Les mesures de fiabilité et de validité n'ont pas encore été publiées pour ces mesures, mais cette étude contribuera à leur évaluation. Les scores de confiance en soi ont été évalués pour 12 procédures SONU. Les scores de connaissances ont été évalués pour deux soins clés de SONU-B, la gestion de l'HPP et la réanimation néonatale (NR) au départ de l'étude et à trois mois après l'intervention mLearning. De plus, les caractéristiques démographiques ont été collectées pour les prestataires des groupes d'intervention et témoins.

Les naissances, les décès maternels, les complications obstétricales et l'exécution des fonctions clés des SONU-B ont été évalués dans les groupes d'intervention et témoins après l'intervention à l'aide d'une application ODK sur smartphone conçue pour cette étude par les chercheurs et testée avec la SDA, dans le cadre d'un examen des procédures pour un futur essai randomisé à plus grande échelle. Les données générées par ODK ont été comparées aux données statistiques collectées manuellement dans les registres des établissements de santé et aux données SNIS rapportées dans le DHIS2.

La faisabilité et l'acceptabilité de la SDA ont été évaluées par le biais d'entretiens qualitatifs semi-structurés avec les utilisateurs de l'application. Les perspectives des principales parties prenantes sur l'utilisation de mLearning plus largement en RDC ont également été évaluées.

Collecte de données

La collecte de données a été menée en parallèle dans les établissements d'intervention et témoins en utilisant les mêmes méthodes au départ et trois mois après l'intervention de formation. Des mesures ont été prises avant la formation des professionnels de santé sur la SDA. Elles comprenaient les informations suivantes:

- Les données démographiques sur les prestataires participants (avant l'intervention)

- L’auto-évaluation du prestataires dans la gestion de 12 procédures SONU
- Les connaissances des prestataires de deux soins SONU-B clés: gestion de l’HPP et RN

Toutes les données ont été recueillies sur papier dans une salle. L'évaluation des connaissances a été fournie par la SDA. Les résultats ont été saisis dans Microsoft Excel, puis transférés et analysés dans SPSS (version 23). Trois mois après l'introduction de la SDA, la confiance en soi et les connaissances ont été mesurées une deuxième fois à l'aide des mêmes instruments de collecte de données.

La chercheuse principale a élaboré deux guides d’entretien qualitatifs pour les deux groupes cibles qualitatifs (utilisateurs da la SDA et parties prenantes clés) en utilisant les domaines de construction théoriques du Cadre de Domaines Théoriques[31] pour orienter les questions. Les entretiens semi-structurés ont été enregistrés sur bande audio par la chercheuse principale auprès de huit utilisateurs de la SDA et de 10 parties prenantes clés après la période d'étude de trois mois. Les utilisateurs de SDA ont été interrogés sur la faisabilité et l'acceptabilité de l'utilisation de la SDA, ainsi que sur les obstacles et les facilitateurs de son utilisation. De même, les parties prenantes clés ont été interrogés sur la faisabilité et l'acceptabilité de l'utilisation de mLearning et de la FC en RDC, ainsi que sur les obstacles et les facilitateurs à la mise en œuvre de la FC.

Les rapports des résultats sur la santé collectés dans les huit établissements avec l’ODK ont été comparés aux données du SNIS et à celles collectées manuellement par la chercheuse principale dans les registres des établissements de santé. Les données ont été collectées manuellement au départ (pour les 3 mois précédant) puis 3 mois après l'intervention afin de permettre une comparaison. L'application ODK a été développée pour cette étude de recherche et pilotée au cours de la période d'étude dans les huit établissements d'intervention et témoins. Les données ODK ont été saisies par les professionnels de santé dans les téléphones mobiles fournis par le projet immédiatement après l’accouchement/l'événement et étaient disponibles en ligne, après téléchargement, pour des consultations depuis n'importe quel endroit.

Analyses statistiques

Des statistiques descriptives résumées ont été analysées sur des données démographiques des participants comprenant l'âge, le sexe, la profession, le niveau d'éducation, le nombre d'années d'expérience, le nombre d'accouchements effectués le mois dernier et l'utilisation antérieure d'un smartphone. S'agissant d'une étude de faisabilité, aucun calcul de puissance n'a été effectué pour choisir la taille de l'échantillon pour l'essai pilote. Cependant, l'équipe chargée de l'étude a orienté la stratégie d'échantillonnage pour atteindre une taille d'échantillon minimale de 30 pour les groupes d'intervention et les groupes témoins afin de soutenir l'utilisation de tests statistiques paramétriques.

Les tests « t » examinaient les différences intra-sujets sur les scores des tests avant et après l'intervention (la variable dépendante étant le score aux tests de confiance en soi et de connaissances au sein des groupes d'intervention et de témoins) et les différences entre les groupes quant à l'évolution de l'auto-évaluation de la confiance en soi et des connaissances (où la variable dépendante était la différence moyenne de changement des scores pour les 2 groupes). Les intervalles de confiance (IC) et l’ampleur de l'effet ont été calculés. Pour tester les facteurs de confusion potentiels, les différences entre les groupes ont été calculées pour examiner le rôle du sexe dans les résultats des tests et le rôle de l'utilisation antérieure du smartphone. Une analyse de variance unidirectionnelle (ANOVA) a été utilisée pour examiner les résultats des tests réalisés par les 3 catégories de professionnels de la santé (infirmières, sages-femmes et médecins) dans les groupes d'intervention et les groupes témoins. Le seuil de signification pour toutes les analyses a été fixé à $p < 0.05$. Toutes les données ont été saisies dans Microsoft Excel et l'analyse a été réalisée à l'aide de SPSS (version 23).

Analyses qualitatives

Le codage des données pour les deux groupes cibles a été effectué de manière déductive par la chercheuse principale, en utilisant les 14 domaines du Cadre de Domaines Théoriques en tant que cadre de codage pour l'analyse de contenu, afin d'interpréter le sens du contenu des données qualitatives.[30–31]

Les réponses des parties prenantes clés ont été mises en correspondance avec ces 8 domaines, ainsi que 2 domaines supplémentaires (rôle social/professionnel et identité, et appréciation des conséquences), car la discussion a pris en compte les problèmes plus généraux et le contexte de la FC en RDC. La portée de l'analyse était limitée aux domaines ci-dessus. Les données

quantitatives et qualitatives ont été interprétées et fusionnées ensemble, en notant à la fois les résultats statistiques quantitatifs et les citations ou thèmes qualitatifs qui corroboraient ou réfutaient les résultats quantitatifs.[27]

RÉSULTATS

Données Quantitatives

Caractéristiques démographiques des participants

L'analyse a inclus 62 prestataires de soins: 32 dans les groupes d'intervention et 30 dans les groupes témoins. Le Tableau 1 montre que les participants comprenaient 26 infirmiers et sages-femmes (81,3%) dans les groupes d'intervention et 20 (66,6%) dans les groupes témoins; les autres participants étaient des médecins (18,8% dans le groupe des établissements d'intervention et 33,3% dans les groupes témoins). L'âge moyen était similaire, 41,3 ans et 44,2 ans dans les groupes d'intervention et témoin respectivement. Il y avait plus de femmes dans les groupes d'intervention (n = 26, 81,3%) que dans les groupes témoins (n=11, 36,7%). Les groupes témoins avaient moins d'expérience d'accouchement que les groupes d'intervention, avec 13 prestataires (43,3%) ayant effectué 5 accouchements ou moins le mois précédent et 10 (33,3%) ayant effectué plus de 10 accouchements, contre 5 (15,6%) et 21 (65,6%), respectivement, des prestataires des groupes d'intervention. De même, 12 prestataires (40,0%) avaient plus de 10 ans d'expérience dans la profession dans les groupes témoins, contre 17 (53,1%) dans les groupes d'intervention. Quatorze prestataires des groupes d'intervention (43,8%) et neuf prestataires des groupes témoins (30,0%) avaient utilisé un smartphone avant l'étude.

Connaissances de base et scores de confiance en soi

Les scores moyens de connaissances pour la prise en charge de l'HPP étaient similaires au départ

TABLEAU 1. Caractéristiques démographiques des participants

	Intervention (n=32)	Témoin (n=30)
**Age (années, moyen)**	41,3	44,2
**Sexe, No. (%)**		
Homme	6 (18,8)	19 (63,3)
Femme	26 (81,3)	11 (36,7)
**Catégorie professionnelle, No. (%)**		
Infirmiers	22 (68,8)	14 (46,6)
Accoucheuses – Sages-Femmes	4 (12,5)	6 (20,0)
Médecins	6 (18,8)	10 (33,3)
**Années d'expérience dans la profession, No. (%)**		
1–5	3 (9,4)	6 (20,0)
6–10	12 (37,5)	12 (40,0)
>10	17 (53,1)	12 (40,0)
**No. d'accouchements le mois précédent, No. (%)**		
0–5	5 (15,6)	13 (43,3)
6–10	6 (18,8)	7 (23,3)
>10	21 (65,6)	10 (33,3)
**Expérience antérieure avec smartphone, No. (%)**		
Oui	14 (43,8)	9 (30,0)
Jamais	18 (56,3)	21 (70,0)

Abréviations: No., nombre

pour les prestataires des groupes d'intervention et témoins à 47,8 (écart type [ET]=16,8) et 47,5 (ET=14,7), respectivement, sur 100 points au total (Tableau 2). Les scores moyens de confiance en soi des SONU-B étaient également similaires au départ dans les groupes d'intervention et témoins à 30,3 (ET=8,7) et 31,4 (ET=10,8), respectivement, sur un total de 48 points. En revanche, les scores moyens de connaissance de base de la RN étaient plus bas chez les prestataires du groupe d'intervention (40,8 ET=17,5) par rapport au groupe témoin (50,9 ET=16,6) (sur 100 points).

Différences des scores de connaissances et de confiance en soi avant et après l'intervention

Nous avons constaté une association significative entre l'intervention de la SDA et les connaissances des prestataires en matière de HPP et de RN, ainsi qu'en ce qui concerne la confiance en soi pour réaliser les procédures SONU-B, trois mois après le début de l'exercice (Tableau 2). L'augmentation moyenne des connaissances sur l'HPP avant et après était statistiquement significativement plus grande dans le groupe d'intervention par rapport au groupe témoin (18,9 ET=14,6 contre 1,6 ET=11,4, respectivement, P<0,001 pour la différence de la différence). De même, l'augmentation moyenne des connaissances sur la RN depuis le pré-test jusqu'au post-test dans le groupe d'intervention était statistiquement plus importante comparée à l'augmentation dans le groupe témoin (16,8 ET=14,6 contre -2,5 ET=16,9 respectivement, avec P<0,001 pour la différence de la différence), malgré des scores de départ plus bas dans le groupe d'intervention. Les scores globaux de confiance en soi pour 12 procédures SONU ont également été significativement améliorés dans les groupes

TABLEAU 2. Différences des scores de connaissances et de confiance en soi, Pré et Post intervention

	Intervention (n=32)	Témoin (n=30)	Différence entre Inter-vention et Témoin	95% IC	*P*	Cohen's d
**Scores connaissances HPP (sur 100)**						
Pré, moyenne (ET)	47,8 (16,8)	47,5 (14,7)	0,3	(−7,8 ; 8,3)	0,95	
Post, moyenne (ET)	66,7 (14,8)	49,1 (15,6)				
Différence pré-post, moyenne (ET)	18,9 (14,6)	1,6 (11,4)	17,4	(10,7 ; 24,0)	<0,001	1,6
95% IC (différence pré-post)	(13,7, 24,2)	(−2,6, 5,8)				
P (différence pré-post)	<0,001	0,46				
Cohen's d	1,2					
**Scores connaissances RN (sur 100)**						
Pré, moyenne (ET)	40,8 (17,5)	50,9 (16,6)	−10,1	(−18,7 ; −1,4)	0,02	−0,6
Post, moyenne (ET)	57,7 (15,3)	48,3 (17,3)				
Différence pré-post, moyenne (ET)	16,8 (14,6)	−2,5 (16,9)	19,4	(11,4 ; 27,4)	<0,001	1,2
95% IC (différence pré-post)	(11,6, 22,1)	(−3,8, 3,8)				
P (différence pré-post)	<0,001	0,42				
Cohen's d	1,0					
**Scores de confiance en soi SONU-B (sur 48)**						
Pré, moyenne (ET)	30,3 (8,7)	31,4 (10,8)	−1,1	(−6,1 ; 3,9)	0,66	
Post, moyenne (ET)	34,0 (8,9)	30,9 (8,7)				
Différence pré-post, moyenne (ET)	3,8 (6,6)	−0,4 (7,2)	4,2	(0,7 ; 7,7)	0,02	0,6
95% IC (différence pré-post)	(1,4, 6,2)	(−8,6, 2,3)				
P (différence pré-post)	0,003	0,74				
Cohen's d	0,4					

Abréviations: SONU-B, soins obstétricaux et néonatals d'urgence de base (SONU-B); IC, intervalle de confiance; RN, réanimation néonatale; HPP, hémorragie post-partum, ET, écart type

TABLEAU 3. Différences des Scores de Confiance en Soi, SONU-B des participants à l'intervention, Pré- et Post-Intervention (sur 4 points)

Procédures SONU-B	Pré-Moyen (ET)	Post-Moyen (ET)	Différence Pré-Post	*P*
AMIU	2,1 (1,3)	3,0 (1,2)	0,88	0,003
D&C	2,7 (1,2)	2,6 (1,3)	−0,03	0,90
Prééclampsie/éclampsie	2,0 (1,2)	2,4 (1,4)	0,41	0,03
GATPA	3,5 (0,7)	3,7 (0,5)	0,22	0,09
Travail prolongé	2,7 (1,1)	3,1 (0,8)	0,47	0,007
Extraction par ventouse obstétricale	1,7 (1,4)	1,8 (1,3)	0,19	0,45
HPP	2,6 (1,2)	3,1 (0,9)	0,50	0,03
Rétention placentaire	2,8 (1,0)	3,1 (1,1)	0,31	0,04
Septicémie maternelle	2,2 (1,2)	2,5 (1,3)	0,34	0,08
RN	3,0 (0,8)	3,0 (0,9)	0,00	1,00
Identification et gestion des signes de danger chez le nourrisson	2,6 (1,0)	2,9 (1,0)	0,31	0,11
Gestion d'infection sévère chez le nourrisson	2,4 (1,1)	2,6 (1,0)	0,19	0,31

Abréviations: AMIU, aspiration manuelle intra-utérine; GATPA, gestion active de la troisième phase de l'accouchement; D&C, dilatation et curetage; RN, réanimation néonatale; HPP, hémorragie post-partum.

d'intervention par rapport à ceux des témoins après 3 mois (différence moyenne de la différence, 4,2 sur 48; IC 95% = 0,7 à 7,7; P=0,02). Des différences significatives dans la confiance en soi des participants à l'intervention ont été trouvées avant et après sur 5 procédures SONU sur 12: aspiration manuelle intra-utérine, prééclampsie/éclampsie, travail prolongé, HPP, et extraction manuelle du placenta (Tableau 3).

Analyse des facteurs de confusion potentiels

En explorant les facteurs de confusion potentiels, la comparaison des scores moyens de connaissance en HPP et en RN par sexe des prestataires dans les groupes d'intervention et de témoins combinés a montré qu'il existait des différences significatives entre les sexes dans le pré-test pour l'HPP ainsi que pour la RN. Cependant, il n'y avait aucune différence entre les hommes et les femmes pour les deux post-tests (Tableau 4). Les scores moyens de connaissances lors du pré-test chez les hommes des groupes d'intervention et témoins étaient statistiquement significativement plus élevés pour les connaissances sur l'HPP et la RN (53,9 ET=13,8 et 53,6 ET=15,9 respectivement) par rapport aux scores des femmes (43,4 ET=15,6 et 40,3 ET=16,9 respectivement) (P=0,008 pour les différences de l'HPP entre hommes et femmes et P=0,003 pour les différences de RN entre hommes et femmes). En revanche, les hommes avaient des scores moyens similaires à ceux des femmes sur les post-tests pour l'HPP et la RN. L'amélioration moyenne des connaissances sur l'HPP avant et après chez les femmes était beaucoup plus importante que chez les hommes (13,8, ET = 17,3 contre 5,7, ET = 11,8, respectivement, avec P = 0,046). De même, l'amélioration moyenne des connaissances sur la RN chez les femmes était statistiquement plus importante comparée à l'amélioration chez les hommes (12,0, ET = 19,0 contre 0,8, ET = 15,7, respectivement, avec P=0,02).

L'analyse des résultats des tests en fonction de l'expérience antérieure dans l'utilisation d'un smartphone a montré des différences significatives entre les scores moyens HPP des pré-tests et des post-tests avec les groupes d'intervention et des groupes témoins combinés. En effet, les personnes ayant déjà utilisé un smartphone ont obtenu des résultats statistiquement significativement supérieurs aux pré et post-tests sur HPP ($P < 0,05$) (Tableau 4). Bien que les personnes ayant eu une expérience avec un smartphone aient obtenu des résultats légèrement supérieurs aux pré et post-tests sur la RN, il n'y avait pas de différence statistiquement significative. De même, il n'y avait pas de différence statistiquement significative dans le changement moyen des connaissances sur l'HPP et la RN, entre pré-test et post-test, entre les personnes expérimentées avec les smartphones et celles qui ne les avaient jamais utilisés.

TABLEAU 4. Différences des Scores Connaissances HPP et RN, Pré- et Post-Intervention (Participants des groupes Intervention et Témoin combinés) Analysés par Sexe et Expérience avec Smartphones

	Hommes (n=25)	Femmes (n=37)	Différence entre Hommes et Femmes	95% IC	*P*	Cohen's d	Jamais utilisé Smartphone (n=39)	Expérience avec Smartphone (n=23)	Différence dans Expérience Smartphone	95% IC	*P*	Cohen's d
**Scores connaissances HPP (sur 100)**												
Pré, moyenne (ET)	53,9 (13,8)	43,4 (15.6)	10,5	(2,8, 18,3)	0,008	0,72	43,0 (14,5)	55,6 (14,6)	12,6	(5,0, 20,3)	0,002	0,87
Post, moyenne (ET)	59,6 (15,3)	57,2 (18,9)	2,5	(−6,6, 11,6)	0,588		53,8 (17,2)	65,6 (15,7)	11,8	(3,0, 20,5)	0,009	0,72
Différence pré-post, moyenne (ET)	5,7 (11,8)	13,8 (17,3)	8,1	(0,1, 16,0)	0,046	0,56	10,9 (17,8)	10,0 (11,6)	0,85	(−7,5, 9,2)	0,84	
95% IC (différence pré-post)	(0,9, 10,6)	(8,0, 19,5)					(5,1, 16,6)	(5,0, 15,0)				
P (différence pré-post)	0,02	<0,001					0,001	<0,001				
Cohen's d	0,37	0,80					0,69	0,66				
**Scores connaissances RN (sur 100)**												
Pré, moyenne (ET)	53,6 (15,9)	40,3 (16,9)	13,3	(4,8, 21,9)	0,003	0,81	43,9 (17,5)	48,7 (18,0)	4,7	(−4,6, 14,0)	0,31	
Post, moyenne (ET)	54,4 (14,8)	52,3 (18,3)	2,1	(-6,7, 10,9)	0,634		51,5 (17,8)	55,9 (15,0)	4,3	(−4,5, 13,2)	0,33	
Différence pré-post, moyenne (ET)	0,8 (15,7)	12,0 (19,0)	11,2	(2,1, 20,4)	0,02	0,66	7,6 (19,4)	7,2 (17,0)	0,40	(−9,4, 10,2)	0,94	
95% IC (différence pré-post)	(−5,7, 7,2)	(5.6, 18.3)					(1,3, 13,9)	(-0,1, 14,6)				
P (différence pré-post)	0,81	<0,001					0,02	0,05				
Cohen's d		0,68					0,43	0,44				

Les tests ANOVA ont été utilisés pour examiner les différences de résultats de test entre les profiles des prestataires en classant tous les participants des groupes d'intervention et témoins en 3 groupes (infirmières, sages-femmes et médecins) (Tableau 5). Les tests ANOVA indiquaient des différences moyennes de groupes statistiquement significatives entre ces 3 profils uniquement pour le pré-test sur la RN. Pour les pré-tests RN et HPP, les médecins avaient les scores les plus élevés, suivis des sages-femmes puis des infirmières. Dans les deux post-tests, les infirmières ont obtenu des scores plus élevés que les sages-femmes, les médecins ayant obtenu les meilleurs résultats. Cette différence reflète positivement la validité interne des instruments de mesure pour distinguer les différences entre les profils des prestataires de soins.

Résultats cliniques

En ce qui concerne les données sur les résultats sur les patients, les chiffres de mortalité néonatale et maternelle ont été recueillis par un examen des registres des données saisies manuellement dans les huit établissements de l'étude. Ces données ont été triangulées avec les données collectées par les téléphones mobiles à l'aide de l'application ODK et des rapports mensuels du SNIS. Les données générées par l'ODK correspondaient bien aux données collectées manuellement des registres. Cependant, les données SNIS rapportées dans le DHIS2 différaient des données ODK et de registres. Les évènements indésirables observés chez les patientes étaient trop peu nombreux pour pouvoir être comparés statistiquement, étant donné le nombre d'établissements et le nombre de mois de la période d'étude, ainsi que la survenue peu fréquente de décès maternel. La collecte de données d'exécution de fonctions clés SONU-B et de complications obstétricales a été testée avec l'ODK, mais n'est pas collectée systématiquement dans les registres des établissements de santé et n'est pas capturée par les rapports SNIS. Par conséquent, ces données n'ont pas pu être comparées. L'ODK n'ayant été introduit qu'au moment de l'intervention (en mai 2017), nous n'avons pas été en mesure de comparer les données ODK avant et après l'intervention.

Données qualitatives

Les données des entretiens ont montré que la SDA et le mLearning peuvent contribuer de manière positive au changement de comportement des prestataires de services SONU-B à travers leur

TABLEAU 5. Différences des Scores Connaissances HPP et RN Pré- et Post-Intervention (Participants des groupes Intervention et Témoin combinés) Analysés par Catégorie Professionnelle des Prestataires des Soins

	Scores, Moyens (ET)						P : Comparaisons Appariés		
	Infirmiers (n=36)	Accoucheuses/ Sages-Femmes (n=10)	Médecins (n=16)	Toutes Catégories (N=62)	F	*P*	Infirmiers–Accoucheuses	Infirmiers–Médecins	Accoucheuses–Médecins
**Pré-tests**									
HPP	44,4 (16,8)	48,8 (14,1)	54,1 (12,3)	47,6 (15,7)	2,197	0,12	—	—	—
RN	41,1 (18,5)	47,5 (11,8)	54,9 (15,5)	45,7 (17,7)	3,747	0,03	—	0,02	—
**Post-tests**									
HPP	56,9 (17,6)	53,2 (17,5)	64,1 (16,7)	58,2 (17,5)	1,428	0,25	—	—	—
RN	53,4 (15,1)	42,8 (21,2)	59,0 (15,6)	53,2 (16,9)	3,046	0,055	—	—	0,04

Abréviations: RN, réanimation néonatale; HPP, hémorragie post-partum; ET, écart type
[a]Signifiant Tukey's HSD (« honestly significant test »).

impact sur plusieurs domaines theoriques.[30–31] Les données montrent que les principaux obstacles au changement de comportement des prestataires lors de l'utilisation de la SDA, de mLearning et de FC sont les carences en ressources liées au contexte et de ressources environnementales, ainsi que le rôle social/professionnel, l'identité, et les appréciations sur les conséquences.[30–31]

Entretiens avec des utilisateurs SDA

Les huit prestataires interrogés ont expliqué comment l'utilisation de la SDA était faisable et acceptable. Ils ont déclaré avoir utilisé l'application à la fois dans des situations d'urgence comme outil de travail (dans le cas de l'HPP, de la RN et de l'aspiration manuelle du contenu utérin) et comme outil d'auto-apprentissage. Ils ont perçu un effet positif sur 7 domaines d'un total de 14, décrit dans le Cadre des domaines théoriques (Tableau 6):

- Connaissances
- Compétences
- Appréciation des capacités/la confiance en soi
- Renforcement
- Intentions
- Emotion
- Mémoire/attention/prise de décision

Ils ont noté qu'il était utile « d'entendre et de voir [l'information] en même temps ». Une personne a déclaré: « Nous avions déjà fait les choses à l'aveuglette avec ce que nous avions appris à l'école et ce n'était pas assez. »

Les répondants ont signalé des changements dans leurs intentions, leurs appréciations quant aux capacités et leurs processus de mémoire/attention/prise de décision, qui les ont amenés à modifier leur façon de prendre en charge les complications obstétricales et néonatales, notamment en surveillant les signes vitaux, en pratiquant le massage utérin et la compression bimanuelle pour l'HPP, en utilisant la traction contrôlée pendant la troisième phase du travail, en administrant des liquides intraveineux et du misoprostol pour l'HPP et en utilisant le partogramme. Un répondant a déclaré: « Cela a changé nos vieilles habitudes ». Les répondants ont discuté des changements observés dans les résultats sur les patients à la suite de l'utilisation de la SDA et de la manière dont cela a renforcé leurs nouvelles pratiques, notamment: « Il n'y a plus de décès dus à l'HPP maintenant ». « Tous les enfants sont sauvés avec l'utilisation du ballon d'Ambu pour la RN. » « Nous voyons maintenant moins de fièvre chez les enfants après la RN lorsque nous donnons des antibiotiques. » « L'HPP disparaît si vous appliquez le contenu de la vidéo. » « Avec des informations dans la vidéo pour la RN, vous voyez le nouveau-né revenir. C'est vraiment encourageant. »

En termes d'émotion, un participant a déclaré: «[Les vidéos] sont amusantes et relaxantes. C'est bon pour éduquer les adultes. Il y a de la variété. » Un autre a déclaré: « Les graphiques animés étaient intéressants. Dans d'autres formations, ils parlent et parlent. » Les autres personnes interrogées ont déclaré avoir consulté fréquemment l'application pour renforcer l'information, à la fois en tant

TABLEAU 6. Résultats des Entretiens qualitatifs avec des utilisateurs SDA (n= 8)

Domaines	Citations Illustratives
Connaissances	« Quand nous entendons et voyons [l'information] en même temps, cela apprend beaucoup. »<br>« Nous avions déjà fait les choses à l'aveuglette avec ce que nous avions appris à l'école et ce n'était pas assez. »
Compétences	« La formation nous a donné les compétences nécessaires pour utiliser l'application SDA. »
Appréciation des capacités/ la confiance en soi	« Nous voyons que le misoprostol est efficace [pour l'HPP]. »<br>« Il n'y a plus de décès dus à l'HPP maintenant. L'HPP disparaît si vous appliquez le contenu de la vidéo. »<br>« Nous voyons maintenant moins de fièvre chez les enfants après la RN lorsque nous donnons des antibiotiques. »<br>« Avec des informations dans la vidéo pour la RN, vous voyez le nouveau-né revenir. C'est vraiment encourageant. »
Renforcement	« Nous avons regardé des vidéos tous les 3 jours environ pendant le temps libre à la maternité [seule] ou avec l'équipe de maternité. Également nous l'avons utilisé lors d'une extraction manuelle du placenta et de l'AMIU. »<br>« On peut regarder la vidéo autant de fois qu'on veut. »
Intentions	« Cela a changé nos vieilles habitudes »<br>« Maintenant, nous prenons les signes vitaux et nous utilisons le partogramme lors de l'accouchement. »<br>« Nous aspirions tous les bébés; maintenant, nous aspirons seulement lorsque nous en avons besoin. »<br>« Avant pour GATPA, nous avons mis le bébé de côté. maintenant, nous plaçons bébé peau à peau et encourageons l'allaitement. »<br>« Avant, nous avons tenu le bébé à l'envers après l'accouchement et nous avons pratiqué le bouche à bouche brutalement si nécessaire. Nous utilisons maintenant le ballon d'Ambu, ce qui donne un bon résultat. Nous avons appris qu'il faut positionner le bébé et le masque pour pouvoir faire de la RN. »<br>« Avant, pour la détresse respiratoire, nous faisions le bouche à bouche et donnions de l'hydrocortisone par voie IM, sans antibiotiques, et nous observions un taux élevé de fièvre. Maintenant, on donne des antibiotiques et on voit moins de fièvre. »<br>« Avant, nous ne faisions pas de massage utérin et n'utilisions pas du misoprostol ou de solutions intraveineuses pour la prise en charge de l'HPP; maintenant, nous utilisons le massage, du misoprostol et des solutions intraveineuses. . . avec de bons résultats. »<br>« Avant, nous administrions du misoprostol sous la langue; maintenant nous le donnons oralement ou par voie rectale. »<br>« Maintenant, nous faisons le massage utérin [en cas d'HPP] et utilisons une sonde urinaire, et nous voyons la contraction de l'utérus. »<br>« Avant, nous repoussions l'utérus au cours de la troisième phase. Maintenant, nous soutenons l'utérus et pratiquons la traction contrôlée sur le cordon ».<br>« Maintenant, en cas de rupture prématurée des membranes, nous administrons des antibiotiques. »
Mémoire / attention / prise de décision	« Apprendre avec l'application est bon, l'apprenant voit l'information, l'entend et peut le faire lui-même. Cela aide les participants à se souvenir des images visuelles ou des informations auditives. »
Contexte et des ressources disponibles	« Seulement deux d'entre nous ont été formés en 2012, mais nous avons besoin de former d'autres personnes et nous avons besoin d'une supervision formative plus souvent.»<br>« La disponibilité du matériel nous aiderait à mieux prendre en charge : uniformes, blouses, bottes, équipement de protection des yeux, tabliers, savon. Nous travaillons avec nos propres vêtements et chaussures, nous risquons de contaminer nos enfants. »<br>« Nous avons besoin de certains matériaux pour bien travailler : de longs gants et de lumière. »<br>« Nous ne gagnons rien. Mettez-vous à notre place. Nous travaillons dur pour rien. »
Emotion	« [Les vidéos] sont amusantes et relaxantes. C'est bon pour enseigner les adultes. Il y a de la variété. »<br>« L'application devrait être plus largement disponible - en pédiatrie et en salle d'opération. »<br>« Les vidéos animées étaient intéressants. Dans d'autres formations, ils parlent et parlent. »<br>« Nous sommes très satisfaits de l'intervention. C'est très encourageant. »

Abréviations: FC, formation continue; RN, réanimation néonatale; HPP, hémorragie post-partum;

qu'outil d'apprentissage et dans diverses urgences obstétricales et néonatales en tant qu'aide au travail.

Le sujet de l'application le plus consulté par les répondants était la gestion de l'HPP (n=8). Les participants ont préféré regarder les vidéos animées par rapport aux fonctionnalités de l'application écrite, et de nombreux prestataires interrogées n'avaient pas consulté les autres fonctionnalités de l'application. Après la vidéo HPP, les autres vidéos les plus fréquemment visionnées portaient sur des sujets liés à la gestion active du troisième stade du travail (n = 5), RN (n = 4), hypertension et la prééclampsie (n = 2), soins du nouveau-né (n = 2), septicémie (n = 1), aspiration manuelle intra-utérine (n = 1), extraction manuelle du placenta (n = 1) et travail prolongé (n = 1).

En ce qui concerne les obstacles à la mise en œuvre des directives SDA et SONU-B, les participants ont cité les conditions de travail et des ressources disponibles, en particulier les conditions inadéquates de l'environnement de travail, le manque d'équipements et de médicaments essentiels, le manque d'électricité et les salaires modiques. Un répondant a déclaré: « La disponibilité du matériel nous aiderait à mieux prendre en charge: uniformes, chemises, chaussures, équipement de protection des yeux, tabliers, savon. Nous travaillons avec nos propres vêtements et chaussures, nous risquons de contaminer nos enfants. » Un autre a déclaré: « Nous avons besoin de certains matériaux pour bien travailler: de longs gants et de lumière. » « Nous ne gagnons rien. Mettez-vous à notre place. Nous travaillons dur pour rien. »

Entretiens avec des parties prenantes clés

Les données des entretiens semi-structurés avec 10 parties prenantes clés ont été mis en correspondance avec les mêmes domaines que ceux notifiés par les utilisateurs de la SDA, plus deux domaines supplémentaires (rôle social/professionnel et identité, et appréciation sur les conséquences) (Tableau 7).[30–31] Les répondants ont soutenu la faisabilité et l'acceptabilité de mLearning et son impact potentiel sur la mortalité maternelle et néonatale. De nombreux répondants ont indiqué que les prestataires n'avaient souvent pas accès à la formation continue et que et que ce sont parfois les mêmes personnes qui sont sélectionnées à plusieurs reprises pour des formations. Un répondant a déclaré: « Les opportunités de participer à une FC sont faibles. L'accès à la FC est limité pour les zones d'accès difficile et les cadres professionnels de ces zones sont moins qualifiés. » Une autre personne interviewée a déclaré: « Le mLearning peut former davantage de personnes à moindre coÃt. »

Les répondants ont indiqué que les formations actuelles sont souvent trop théoriques, qu'elles ne concernent pas nécessairement le travail quotidien des professionnels de santé, que souvent elles ne présentent aucun intérêt et qu'elles ne permettent pas d'améliorer les connaissances, les compétences ou de faire évoluer les appréciations quant aux capacités ou aux intentions de changement de comportement. Un répondant a déclaré: « En règle générale, les compétences du personnel en matière de formation continue sont peu développées. » Un autre a déclaré: « Nous devons modifier les approches en matière de formation pour adopter des méthodes facilitant l'apprentissage. Les approches ne sont pas adaptées à l'époque actuelle, utilisant des modules écrits et des conférences. Les gens ne lisent pas les modules. » Un responsable de service a déclaré: « Les formations par des conférences sont trop théoriques; les notions essentielles ne sont pas maîtrisées et les participants ne sont pas en mesure d'appliquer ces connaissances à un cas particulier. »

Pour lutter contre l'accès peu fréquent à la formation ou à des possibilités d'apprentissage autodirigé, mLearning était cité comme approche qui permet d'offrir la possibilité de renforcer l'apprentissage et d'être plus intéressant (processus d'émotion, de mémoire/d'attention/de prise de décision): « Les nouvelles technologies fascinent les gens et ils veulent essayer. Cela répond à un besoin ou à un désir d'apprendre. » « Les nouvelles technologies doivent être encouragées, en particulier dans les régions isolées. » « L'audiovisuel est plus intéressant et peut être expérimenté seul ou en groupe. Les approches doivent passer des informations écrites à des options d'auto-apprentissage interactives. »

En ce qui concerne les obstacles à mLearning ou à la FC, les réponses étaient centrées sur le domaine des appréciations sur les conséquences et en particulier sur le manque d'incitations ou d'exigences pour la FC, et le manque général de capacités nationales et régionales de planification et de suivi de la FC. Une personne a déclaré: « Il n'y a pas de lien entre la formation continue et la progression de carrière. » D'autres ont déclaré: « La formation continue doit répondre à un besoin, combler un vide et conduire à un changement de statut d'emploi ou à un changement concret. » « Nous avons besoin d'un système d'accréditation pour la FC, afin que les gens suivent la FC avec un plan systématique de cours requis pour différents domaines. » Un répondant a déclaré: « mLearning devrait être associé au suivi et à la supervision post-formation et être lié aux contrats de performance. » Un autre a dit: « Nous devons encourager les prestataires à faire mieux. » Les personnes interrogées ont également noté que de nombreuses carences étaient centrées dans le domaine des ressources, en particulier en raison du contexte, qui est pour beaucoup à la base du manque de soins de qualité, le manque de redevabilité pour les mauvaises pratiques, une rémunération insuffisante pour les professionnels de santé, le manque de médicaments, d'équipements et de supervision. Un répondant a souligné les différences entre ce que les gens apprennent dans les FC et la réalité de

TABLEAU 7. Résultats des Entretiens qualitatives avec des parties prenantes clés (n=10)

Domaines	Citations Illustratives
Connaissances	« Les formations sont théoriques via des conférences. Ils enseignent trop de choses en même temps et se précipitent à travers le matériel. Les apprenants ont du mal à hiérarchiser leurs priorités et partent dans la confusion; pendant ce temps, les notions essentielles ne sont pas maîtrisées et ils ne sont pas capables d'appliquer les connaissances à un cas particulier. Les prestataires ont déjà été formés, mais c'est comme s'ils n'avaient jamais été formés. Il serait préférable d'avoir plus de formation pratique. »<br>« Les formations par des conférences sont trop théoriques ; les notions essentielles ne sont pas maîtrisées et les participants ne sont pas en mesure d'appliquer ces connaissances à un cas particulier. »<br>« La FC doit intégrer les principes d'apprentissage des adultes et doit être continue / régulière. »<br>« Les hôpitaux / prestataires sont souvent soumis à des formations qu'ils n'ont pas planifiées. . . ils devraient être inclus dans la planification en réponse aux lacunes / besoins prioritaires. »<br>« Nous devons modifier les approches en matière de formation pour adopter des méthodes facilitant l'apprentis-sage. Les approches utilisées ne sont pas adaptées à l'époque actuelle, utilisant des modules écrits et des conférences. Les gens ne lisent pas les modules. »<br>« SDA rend l'apprentissage opérationnel. »
Compétences	« En règle générale, les compétences du personnel en matière de formation continue sont peu développées. »<br>« Les soins sont devenus mécanisés et basés sur des protocoles mémorisés, de sorte que les [agents de santé] ont du mal à analyser les situations. »<br>« Les prestataires ont pu mettre en pratique de nouvelles choses apprises [de la SDA] telles que la compression bimanuelle pour l'HPP. »<br>« La FC devrait être menée dans des conditions de travail réelles pour combler l'écart entre ce que l'on sait et ce que l'on fait. »<br>« MLearning enseigne également aux gens comment utiliser la technologie. »
Rôle social/professionnel et identité	« [Les agents de santé] utilisent ce dont ils disposent pour traiter les patients, mais ils ne vont pas chercher quelque chose ou obtenir des informations supplémentaires. »<br>« On doit créer un moyen pour que les gens puissent partager des informations. Peut-être former un club (à animer avec un animateur / encadreur). Si le programme est personnalisé, c'est encore mieux - obtenez des points, obtenez un certificat [doit être lié à l'employeur]. »
Appréciation des capacités/ la confiance en soi	« Imposer la FC n'a qu'une valeur limitée, quand le patron n'est pas là ... ils ne le feront pas. »<br>« Les inspirer aux avantages de la FC serait plus motivant que des sanctions. »
Appréciation sur les conséquences	« Pas de responsabilité liée à une faute professionnelle.»<br>« Les réglementations / exigences de la FCsont les domaines dans lesquels le pays doit se rendre pour apporter un changement. »<br>« [Les employeurs ou] le ministère devraient disposer de la capacité de suivi de la formation continue afin de pouvoir identifier les personnes ayant besoin d'une formation et d'une formation nécessaire. »<br>« Il n'y a pas de lien entre la formation continue et la progression de carrière. » « La formation continue doit répondre à un besoin, combler un vide et conduire à un changement de statut d'emploi ou à un changement concret. »
Renforcement	« MLearning est intéressant pour renforcer l'apprentissage, car l'outil est disponible à tout moment et le prestataire peut afficher les informations plusieurs fois. »<br>« Idéal si un [encadreur] suit le processus pour enrichir le procussus [donner des informations rétroactives] et assurer que les besoins sont couverts dans l'application] et répondre aux questions. »<br>« La SDA devrait être disponible dans toutes les zones de santé avec supervision après la formation. . . cela diminuerait la mortalité maternelle et néonatale. »
Intentions	« Pour les zones reculées, l'apprentissage en ligne et autodirigé est intéressant et doit déterminer s'ils (profes-sionnels de santé) acquièrent des compétences. Ils peuvent être certifiés et mettre en place un système pour qu'ils soient encouragés à le faire. »<br>« Les nouvelles technologies devraient être encouragées, en particulier dans les régions éloignées [sans bibliothèque ni spécialiste]. »
Mémoire / attention / prise de décision	« Les images permettent aux gens d'apprendre avec plus de stimulation et d'attention. La mémoire visuelle peut fixer la mémoire des informations plus longtemps. »<br>« Quand le temps passe après une formation. . . tu oublies." "Si les gens sont motivés, ils retiendront peut-être mieux les informations. »
Contexte et des ressources disponibles	« De meilleures conditions de travail [salaires, supervision, environnement] pousseraient les gens. »<br>« Les défis sont liés à la logistique, l'électricité et les équipements. »<br>« Il faut améliorer l'environnement de travail, cela motive ou démotive les gens. »

Continued

TABLEAU 7. Continued

Domaines	Citations Illustratives
	« Différence entre ce que les gens apprennent et la réalité de l'environnement. »<br>« Les opportunités de participer à une FC sont faibles. L'accès à la FC est limité pour les zones d'accès difficile et les cadres professionnels de ces zones sont moins qualifiés. »<br>« Pour la FC, les programmes / partenaires choisissent le sujet et peuvent répéter les sujets déjà couverts ; les mêmes personnes vont toujours aux formations et sont absentes du travail. »<br>« Les prestataires sont absents du travail pendant les formations à des intervalles fréquents pendant de longues périodes. La longue durée est liée au fait de ne pas connaître le profil des travailleurs qui seront formés et de ne pas connaître les lacunes en matière d'apprentissage, de sorte que les formateurs les forment à tout ce qui concerne l'approche « filet de pêche » (tout rassembler). »<br>« Pas de politique claire de la FC. Qui choisit les sujets ? Et il faut définir qui participe et ce que l'on en tire. Nous avons besoin d'une politique claire, la formation doit être programmée et budgétisée. . . afin que les employeurs reconnaissent la formation et la planifient / budgètisent pour leur établissement. »<br>« Le mLearning peut former davantage de personnes à moindre coÃt. »
Emotion	« Voir les images est plus intéressant que parler théoriquement. »<br>« Formation en ligne - les personnes sont prêtes à travailler pour maîtriser de nouvelles choses, ils se sentent valorisées et désireuses de s'améliorer. »<br>« Les nouvelles technologies fascinent les gens et ils veulent essayer. Cela répond à un besoin ou à un désir d'apprendre. »<br>« Les nouvelles technologies doivent être encouragées, en particulier dans les régions isolées. »<br>« L'audiovisuel est plus intéressant et peut être expérimenté seul ou en groupe. Les approches doivent passer des informations écrites à des options d'auto-apprentissage interactives. »

Abréviations: FC, formation continue; SDA Safe Delivery App

l'environnement: « les défis sont liés à la logistique, l'électricité et les équipements. »

Triangulation de données quantitatives et qualitatives

Les entretiens qualitatifs ont révélé de nombreux avantages positifs pour l'utilisation de la SDA et de mLearning dans le contexte de la RDC, en particulier pour ce qui est de mettre à la disposition des professionnels de santé des directives mondiales SONU-B actualisées et fondées sur des preuves empiriques, via une application stimulante de mLearning. L'accès à l'information a été noté comme particulièrement critique pour ceux qui n'ont souvent aucune possibilité d'apprentissage, tels que les personnes habitant dans les zones reculées et les cadres professionnels de niveau inférieur. Les personnes interrogées ont toutefois noté que même pour les personnes formées dans le passé, la SDA et mLearning offrent la possibilité d'acquérir des connaissances et des compétences importantes et de modifier les comportements en utilisant une approche plus moderne et plus intéressante qui plaise à tout le personnel de santé. Cela renforce nos résultats quantitatifs en ce qui concerne l'amélioration significative des scores de connaissances et de confiance en soi (qui reflète directement les 2 domaines de connaissances et d'appréciation sur les capacités) pour les 3 categories de professionnels de santé (médecins, infirmiers, sages-femmes) après 3 mois d'utilisation de la SDA. L'utilisation antérieure du smartphone n'a eu aucune incidence sur les augmentations des scores, ce qui renforce le fait que mLearning peut être utilisé pour former n'importe quel professionnel de santé.

Les réponses qualitatives des principaux parties prenantes clés en RDC ont en outre permis de mieux comprendre les obstacles à l'apprentissage par mLearning et à la FC, tels que les obstacles environnementaux et contextuels et le manque de ressources (Tableau 7). Le contexte des politiques de la santé, notamment le manque de redevabilité et des mesures incitatives (appréciations sur les conséquences) et le manque d'identité professionnelle, renforcent mutuellement le contexte environnemental précaire et les ressources insuffisantes. Cela contribue considérablement à une mauvaise qualité des soins souvent dispensés par les prestataires de soins et au taux élevé de mortalité des mères et des nouveau-nés.

DISCUSSION

L'étude montre que l'apprentissage via la est faisable et acceptable pour les professionnels de santé

dans le contexte de la RDC. Notre échantillon d'intervenants clés en RDC montre que le mLearning est faisable, acceptable et qu'il peut constituer une solution aux problèmes rencontrés par les prestataires de soins pour accéder à des ressources d'apprentissage actualisées dans des environnements difficiles à atteindre. Un essai pilote avec la version française de SDA dans le centre-est de la RDC a entraîné une amélioration significative des scores de connaissances des professionnels de santé pour la gestion de l'HPP et des RN et des scores de confiance en soi en SONU-B chez les participants des établissements d'intervention par rapport aux participants témoins, indépendamment d'une utilisation antérieure de smartphone ou du profil professionnel du prestataire.

Cette étude a confirmé les conclusions de Lund et al.[14] concernant l'effet significatif de l'utilisation de SDA avec des prestataires qualifiés des soins maternels et néonataux en Éthiopie en termes d'amélioration significative des connaissances et des compétences des professionnels de santé en matière de réanimation néonatale et d'une réduction non significative de 24% de la mortalité périnatale. La recherche présentée dans cet article n'a pas testé les scores de compétences SONU-B, étant donné la nature réduite de l'étude de faisabilité. De même, cette recherche n'a pas permis de déterminer l'impact de la SDA sur les résultats de santé sur les patientes avec suffisamment de puissance, compte tenu de la taille réduite de l'échantillon et de la courte durée de l'étude, associées à la faible fréquence des décès maternels. Cependant, l'équipe de recherche a évalué la faisabilité des procédures d'étude pour une future étude plus vaste et plus importante en RDC.

Bien que la recherche n'ait pas été en mesure de démontrer les deux besoins essentiels en matière d'essais mHealth dans les pays à revenu faible ou intermédiaire identifiés dans les revues systématiques, [21–23] à savoir les résultats sur les patients et les essais à long terme, cette étude a évalué la faisabilité, l'acceptabilité et l'efficacité potentielle de l'utilisation de la SDA pour améliorer la qualité de SONU B dans le plus grand pays d'Afrique francophone. L'étude propose également un moyen potentiel de remédier au problème de l'accès insuffisant à une formation actualisée, basé sur les preuves empiriques, des documents de référence pour les agents de santé dans les zones d'accès difficile et pour les cadres d'agents de santé qui manquent souvent les opportunités de formation en RDC. Les avantages de la SDA sont les suivants: cet outil peut être compris de façon indépendante, il est disponible dans de nombreuses langues, en « source ouverte » (open source) et son téléchargement est gratuit. De plus, une fois installé sur l'appareil mobile, l'application n'a pas besoin de connexion à un réseau internet pour fonctionner.

La formation conventionnelle des prestataires qualifiés des soins maternels et néonatals en SONU s'est révélée efficace pour améliorer les résultats des soins de santé.[11,32,33] Cependant, les agents de santé dans des environnements difficiles à atteindre ne sont souvent pas en mesure de participer à de telles formations et ne peuvent accéder à d'autres ressources pour l'auto-apprentissage.[10,17,18] Les revues systématiques sur la santé mobile (mHealth) montrent que les applications de téléphonie mobile sont de plus en plus utilisées dans les pays à revenu faible ou intermédiaire pour diffuser des informations aux agents de santé.[34] D'autres études pilotes ont montré que les stratégies d'apprentissage mLearning associées étaient potentiellement aussi efficaces que les stratégies de formation traditionnelles.[20] Ces résultats rejoignent également les résultats d'autres études selon lesquelles l'utilisation d'outils électroniques est considérée comme une opportunité pour'améliorer la qualité des soins des agents de santé, avec des effets sur la motivation[35], l'auto-efficacité[36] et l'enthousiasme.[37] Le Cadre des Domaines Théoriques propose des domaines d'influence ou constructions pour le changement de comportement des agents de santé qui reflètent ces concepts, avec des domaines tels que la conviction sur les capacités, les intentions et les émotions en tant que déterminants essentiels du changement de comportement.[30–31]

Les leçons tirées de cette étude de faisabilité pour améliorer les procédures d'études futures sont les suivantes. Dans un premier temps, elles suggèrent que les essais futurs de mLearning en RDC doivent bénéficier d'un moyen supplémentaire de collecte de données sur la mortalité et d'autres données critiques des SONU-B, comme l'utilisation de l'instrument de collecte de données ODK conçu à cet effet ou du personnel d'étude dédié à la collecte de données sur place. Les chercheurs ont constaté que les données recueillies au moyen d'un examen manuel des registres des établissements de santé et des données ODK recueillies quotidiennement par téléphone mobile étaient comparables, mais différaient des rapports mensuels du SNIS. Bien que ce ne soit pas la question principale de notre recherche, les données des entretiens ont révélé des explications possibles aux divergences dans les données recueillies auprès de ces différentes sources. Les entretiens ont mentionné notamment la sous-déclaration

de la mortalité dans le SNIS par les prestataires de soins pour diverses raisons, comme la compilation incomplète des données du SNIS en s'appuyant sur certains registres (maternité) plutôt que tous les registres d'hôpitaux associés, dans lesquels les décès maternels ou néonataux seraient enregistrés (services d'urgence, de gynécologie et de pédiatrie, par exemple), ou l'omission dans la notification des décès, les données SNIS n'étant collectées que mensuellement et pouvant être enregistrées rétrospectivement. Il semble que la sous-déclaration des effets indésirables est réalisée de façon délibérée, par crainte d'une sanction par les autorités sanitaires ou pour des raisons financières. De tels facteurs compliquent alors la mesure des résultats dans une éventuelle étude en utilisant uniquement les données SNIS en RDC. Il conviendrait donc de prendre certaines mesures correctives pour améliorer la qualité des données sur la mortalité.

Les recherches futures pourraient également tirer parti de l'utilisation de la version récemment mise à jour de la SDA, qui intègre des fonctionnalités supplémentaires permettant de mesurer l'apprentissage et de motiver l'utilisateur individuel grâce à des fonctionnalités identiques à celles du jeu, dans lesquelles les utilisateurs doivent gagner un certain nombre de points pour pouvoir passer au niveau d'apprentissage suivant. Une fois qu'ils ont obtenu le meilleur score, ils sont certifiés. Ces fonctionnalités pourraient augmenter de manière importante les avantages de l'intervention de la SDA en offrant des incitations et des récompenses, en encourageant la motivation, en abordant ainsi des domaines tels que l'émotion, le renforcement et la conviction des capacités. Toutefois, ces fonctionnalités s'appuieraient sur Internet et la bande passante, qui restent inaccessibles aux individus dans de nombreux contextes en raison de mauvaises connexions ou de manque d'accès financier, malgré l'augmentation rapide de l'utilisation des communications sans fil dans de nombreux pays en développement.[34]

Les conséquences de cette étude sont les suivantes: la SDA et d'autres interventions de mLearning améliorent la capacité des prestataires de soins à fournir des soins de meilleure qualité pendant les urgences obstétriques et néonatales, ainsi que de meilleurs soins obstétricaux et néonatals de routine. En raison de l'évolution des politiques de formation continue en RDC ainsi que le nombre important de situations dans des contextes similaires, il convient d'envisager l'intégration de mLearning comme approche de formation et d'aide au travail pour SONU-B afin de réduire la mortalité maternelle et néonatale, ainsi que l'intégration des outils de mLearning pour d'autres priorités et problèmes de santé émergents. Les défis de la mise en œuvre de soins SONU-B de qualité, posés par des écarts dans le contexte environnemental et les ressources, ainsi que par les faiblesses de l'environnement réglementaire et de redevabilité, doivent être pris en compte et traités conjointement avec d'autres mesures programmatiques telles que des initiatives d'amélioration de la qualité visant à cibler les faiblesses du système de santé.

Limites

La taille de l'échantillon de cette étude a limité les résultats en réduisant la puissance de l'étude. En termes de limites de conception, huit établissements de santé ont été randomisées dans cette étude, plutôt que des individus, afin d'éviter la diffusion des informations du groupe d'intervention au groupe témoin.[14] Les chercheurs ont relevé des disparités entre les groupes d'intervention et les groupes témoins en termes de sexe, de composition des categories professionnelles, ainsi que par rapport à l'expérience antérieure des prestataires avec des accouchements. De plus, il était impossible de cacher les groupes d'intervention et témoins en raison de la nature de l'intervention, ce qui augmente le risque de biais d'information. Il est toutefois possible que certains participants du groupe témoin aient eu accès à la SDA dans des établissements d'intervention, étant donné que les installations se trouvaient toutes dans une approximation géographique relativement proche. Parmi les autres limitations, mentionnons le fait que l'équipe de l'étude n'a pas été en mesure de suivre systématiquement l'utilisation de la SDA au cours de l'étude, cette capacité n'étant pas complètement développée au moment de l'intervention. L'accès à ces données enrichirait les résultats et l'analyse de l'association entre l'utilisation de la SDA et l'évolution des mesures dans les recherches futures.

CONCLUSION

Les méthodes qualitatives et quantitatives de cette recherche ont montré que l'application SDA et le mLearning sont faisables et acceptables pour les professionnels de santé et les principaux parties prenantes clés en RDC, le plus grand pays Africain francophone. L'utilisation de la SDA a été associée à une connaissance accrue des professionnel de santé sur la prise en charge de l'HPP et la RN trois mois après l'introduction de

l'application, ainsi qu'à une plus grande confiance en soi des professionnels de santé pour la prise en charge des urgences obstétricales et néonatales. Ces résultats s'inscrivent dans les conclusions de nombreuses études récentes sur la santé mobile dans les pays à faible revenu où la qualité des soins est compromise par le manque de programmes de formation continue entre plusieurs factors.

Remerciements: Nous remercions le Ministère de la Santé Publique, RDC; The Maternity Foundation; Drs. Stine Lund et Bjarke Sorensen, Université de Copenhague; Dr. Peter Johnson, Jhpiego; Dr Martina Mueller et Rebeca Mueller, Medical University of South Carolina; Steven Fountain et Chris Kalonji, IMA World Health; et Apolline Foedit, Institut des Hautes Etudes Internationales et du Développement (IHEID), Genève; et Alexandre Delamou, Centre National de Formation et de Recherche en Santé Rurale de Maferinyah. Dédié à Francine Mantudulua.

Financement: IMA World Health.

Intérêts concurrents: Aucun déclaré.

BIBLIOGRAPHIE

1. Rowe AK, de Savigny D, Lanata CF, Victora CG. How can we achieve and maintain high-quality performance of health workers in low-resource settings? *Lancet*. 2005;366(9490):1026–1035. CrossRef. Medline
2. Sorensen BL, Rasch V, Massawe S, Nyakina J, Elsass P, Nielsen BB. Advanced Life Support in Obstetrics (ALSO) and post-partum hemorrhage: a prospective intervention study in Tanzania. *Acta Obstet Gynecol Scand*. 2011;90(6):609–614. CrossRef. Medline
3. Maternal mortality. World Health Organization website. http://www.who.int/mediacentre/factsheets/fs348/en/. Published November 2016. Accessed February 22, 2018.
4. United Nations Inter-agency Group for Child Mortality Estimation (UN IGME). *Levels and Trends in Child Mortality: Report 2017*. New York: United Nations Children's Fund; 2017. https://www.unicef.org/publications/files/Child_Mortality_Report_2017.pdf. Accessed February 22, 2018.
5. World Health Organization (WHO). *Trends in Maternal Mortality: 1990 to 2015*. Geneva: WHO; 2015. https://www.who.int/reproductivehealth/publications/monitoring/maternal-mortality-2015/en/. Accessed February 22, 2018.
6. Ministère du Plan et Suivi de la Mise en œuvre de la Révolution de la Modernité (MPSMRM); Ministère de la Santé Publique (MSP); ICF International. *Democratic Republic of Congo Demographic and Health Survey 2013-14: Key Findings*. Rockville, MD: MPSMRM, MSP, and ICF International; 2014. https://dhsprogram.com/pubs/pdf/SR218/SR218.e.pdf. Accessed February 22, 2018.
7. Kalyan G, Vatsa M. Neonatal nursing: an unmet challenge in India. *Indian J Pediatr*. 2014;81(11):1205–1211. CrossRef. Medline
8. Moran NF, Naidoo M, Moodley J. Reducing maternal mortality on a countrywide scale: the role of emergency obstetric training. *Best Pract Res Clin Obstet Gynaecol*. 2015;29(8):1102–1118. CrossRef. Medline
9. Cancedda C, Farmer PE, Kyamanywa P, et al. Enhancing formal educational and in-service training programs in rural Rwanda: a partnership among the public sector, a nongovernmental organization, and academia. *Acad Med*. 2014;89(8):1117–1124. CrossRef. Medline
10. Mirkuzie AH, Sisay MM, Bedane MM. Standard basic emergency obstetric and neonatal care training in Addis Ababa; trainees reaction and knowledge acquisition. *BMC Med Educ*. 2014;14(1):201. CrossRef. Medline
11. Ameh C, Adegoke A, Hofman J, Ismail FM, Ahmed FM, van den Broek N. The impact of emergency obstetric care training in Somaliland, Somalia. *Int J Gynaecol Obstet*. 2012;117(3):283–287. CrossRef. Medline
12. Figueras A, Narváez E, Valsecia M, et al. An education and motivation intervention to change clinical management of the third stage of labor–the GIRMMAHP Initiative. *Birth*. 2008;35(4):283–290. CrossRef. Medline
13. Say L, Chou D, Gemmill A, et al. Global causes of maternal death: a WHO systematic analysis. *Lancet Glob Health*. 2014;2(6):e323–e333. CrossRef. Medline
14. Lund S, Boas IM, Bedesa T, Fekede W, Nielsen HS, Sørensen BL. Association between the Safe Delivery app and quality of care and perinatal survival in Ethiopia: a randomized clinical trial. *JAMA Pediatr*. 2016;170(8):765–771. CrossRef. Medline
15. Maternity Foundation website. http://www.maternity.dk/our-work/. Accessed December 22, 2017.
16. World Health Organization (WHO). *The World Health Report 2006: Working Together for Health*. Geneva: WHO; 2006. http://www.who.int/whr/2006/whr06_en.pdf. Accessed February 22, 2018.
17. Hudspeth J, Curry CL, Sacks Z, Surena C. Continuing professional development in low-resource settings: Haiti as example. *Ann Glob Health*. 2015;81(2):255–259. CrossRef. Medline
18. Hosey KN, Kalula A, Voss J. Establishing an online continuing and professional development library for nurses and midwives in East, Central, and Southern Africa. *J Assoc Nurses AIDS Care*. 2016;27(3):297–311. CrossRef. Medline
19. Sondaal SFV, Browne JL, Amoakoh-Coleman M, et al. Assessing the effect of mHealth interventions in improving maternal and neonatal care in low- and middle-income countries: a systematic review. *PLoS One*. 2016;11(5):e0154664. CrossRef. Medline
20. Nilsson C, Sørensen BL, Sørensen JL. Comparing hands-on and video training for postpartum hemorrhage management. *Acta Obstet Gynecol Scand*. 2014;93(5):517–520. CrossRef. Medline
21. Lee SH, Nurmatov UB, Nwaru BI, Mukherjee M, Grant L, Pagliari C. Effectiveness of mHealth interventions for maternal, newborn and child health in low- and middle-income countries: systematic review and meta-analysis. *J Glob Health*. 2016;6(1):010401. CrossRef. Medline
22. Tomlinson M, Rotheram-Borus MJ, Swartz L, Tsai AC. Scaling up mHealth: where is the evidence? *PLoS Med*. 2013;10(2):e1001382. CrossRef. Medline
23. Labrique AB, Vasudevan L, Kochi E, Fabricant R, Mehl G. mHealth innovations as health system strengthening tools: 12 common applications and a visual framework. *Glob Health Sci Pract*. 2013;1(2):160–171. CrossRef. Medline
24. Francis JJ, O'Connor D, Curran J. Theories of behaviour change synthesised into a set of theoretical groupings: introducing a thematic series on the theoretical domains framework. *Implement Sci*. 2012;7(1):35–35. CrossRef. Medline
25. Pilot and feasibility trials. CONSORT website. http://www.consort-statement.org/extensions/overview/pilotandfeasibility. Accessed March 20, 2018.
26. Creswell JW, Plano Clark VL. *Designing and Conducting Mixed Methods Research*. 2nd ed. Thousand Oaks, CA: SAGE Publications Inc.; 2011.
27. Cleary M, Horsfall J, Hayter M. Data collection and sampling in qualitative research: does size matter? *J Adv Nurs*. 2014;70(3):473–475. CrossRef. Medline
28. Access to Primary Health Care Project. IMA World Health website. https://imaworldhealth.org/dfid/. Accessed February 22, 2018.

29. Hesse-Biber SN, Leavy PL. *The Practice of Qualitative Research*. 2nd ed. Thousand Oaks, CA: SAGE Publications Inc.; 2011.

30. Atkins L, Francis J, Islam R, et al. A guide to using the Theoretical Domains Framework of behaviour change to investigate implementation problems. *Implement Sci*. 2017;12(1):77. CrossRef. Medline

31. Cane J, O'Connor D, Michie S. Validation of the theoretical domains framework for use in behaviour change and implementation research. *Implement Sci*. 2012;7:37. CrossRef. Medline

32. Grady K, Ameh C, Adegoke A, et al. Improving essential obstetric and newborn care in resource-poor countries. *J Obstet Gynaecol*. 2011;31(1):18–23. CrossRef. Medline

33. Ellard DR, Shemdoe A, Mazuguni F, et al; ETATMBA Study Group. Can training non-physician clinicians/associate clinicians (NPCs/ACs) in emergency obstetric, neonatal care and clinical leadership make a difference to practice and help towards reductions in maternal and neonatal mortality in rural Tanzania? The ETATMBA project. *BMJ Open*. 2016;6(2): e008999. CrossRef. Medline

34. Agarwal S, Perry HB, Long LA, Labrique AB. Evidence on feasibility and effective use of mHealth strategies by frontline health workers in developing countries: systematic review. *Trop Med Int Health*. 2015;20(8):1003–1014. CrossRef. Medline

35. Zakane SA, Gustafsson LL, Tomson G, et al. Guidelines for maternal and neonatal "point of care": needs of and attitudes towards a computerized clinical decision support system in rural Burkina Faso. *Int J Med Inform*. 2014;83(6):459–469. CrossRef. Medline

36. Walker DM, Holme F, Zelek ST, et al. A process evaluation of PRONTO simulation training for obstetric and neonatal emergency response teams in Guatemala. *BMC Med Educ*. 2015;15(1):117. CrossRef. Medline

37. Taekman JM, Foureman MF, Bulamba F, et al. A novel multiplayer screen-based simulation experience for African learners improved confidence in management of postpartum hemorrhage. *Front Public Health*. 2017;5:248. CrossRef. Medline

Revu par les pairs

Reçu : juilllet 30, 2018 ; **Accepté :** novembre 6, 2018

© Bolan et al. This is an open-access article distributed under the terms of the Creative Commons Attribution License, which permits unrestricted use, distribution, and reproduction in any medium, provided the original author and source are properly cited. To view a copy of the license, visit http://creativecommons.org/licenses/by/4.0/. When linking to this article, please use the following link: http://ghspjournal.org/lookup/suppl/doi:10.9745/GHSP-D-18-00275/-/DCSupplemental
